# Supplementary material for: First-trimester exposure to benzodiazepines and risk of congenital malformations in offspring: A population-based cohort study in South Korea
Source: PLoS Med. 2022 Mar 2;19(3):e1003945. doi: 10.1371/journal.pmed.1003945 (PMC8926183; doi:10.1371/journal.pmed.1003945)
Supplement: S1 Table — (DOCX) [file pmed.1003945.s002.docx]

**S1 Table.** Codes used to define the inclusion/exclusion criteria, exposures, outcomes of interest, maternal comorbidities, and concomitant medications

| **Categories** | **Codes** |
| --- | --- |
| **Inclusion criteria** | **HIRA procedure codes** |
| Delivery | R3131-R3148, R4351-R4362, R4380, R4507-R4520, R5001-R5002, RA311-RA318, RA361-RA362, and RA380-RA434 |
| **Exclusion criteria** | **ICD-10 or ATC codes** |
| Teratogenic/genetic syndromes, microdeletions, and chromosomal abnormalities | D821, P350-P352, P371, Q619, Q751, Q754, Q771-Q772, Q780, Q796, Q85, Q861-Q869, Q87, Q90-Q92, Q930-Q939, and Q95-Q99 |
| Known teratogens | Antineoplastic agent (L01), warfarin (B01AA03), lithium (N05AN), systemic retinoids (D10BA and D05BB), misoprostol (A02BB01, G02AD06, and M01AE56), thalidomide (L04AX02, L04AX04, and L04AX06), androgens (G03B, G03E, and G03XA), and tetracycline derivative (J01AA) |
| **Exposures** | **ATC codes** |
| Benzodiazepines | N03AE, N05BA, and N05CD |
| **Outcomes of interest** | **ICD-10 codes** |
| Major congenital malformations |  |
| Nervous system | Q00-Q07 |
| Eye | Q100, Q104, Q106-Q109, Q11-Q12, Q130-Q134, Q136-Q139, and Q14-Q15 |
| Ear, face, and neck | Q16, Q176-Q178, Q183, and Q188 |
| Heart defects | Defects of cardiac chambers and connections (Q20); cardiac septal defects (Q21); pulmonary and tricuspid valve defects (Q22); aortic and mitral valve defects (Q23); other heart defects (Q24); defects of the great arteries (Q25); and defects of the great veins (Q260, Q262-Q269) |
| Respiratory system | Q300, Q321-Q329, Q330, Q332-Q335, Q337-Q339, and Q34 |
| Oral clefts | Q35-Q37 |
| Digestive system | Tongue, mouth, and pharynx (Q380, Q383-Q389); oesophagus (Q39); upper alimentary tract (Q402-Q409); small intestine (Q41); large intestine (Q42); other malformations of the intestine (Q431-Q439); gallbladder, bile ducts and liver (Q44); other malformations of the digestive system (Q45); and diaphragmatic hernia (Q790) |
| Abdominal wall defects | Q792-Q793 and Q795 |
| Urinary system | Q60, Q611-Q619, Q620-Q626, Q628-Q629, Q630-Q632, Q634-Q639, Q64, and Q794 |
| Genital organs | Q50-Q51, Q520-Q522, Q524, Q526, Q528-Q529, and Q54-Q56 |
| Limb | Q660-Q661, Q679, Q681-Q682, Q686-Q689, and Q70-Q74 |
| Other malformations | Q750, Q77, Q782-Q788, Q80-Q81, Q820-Q824, Q826-Q829, Q860, Q890, and Q893-Q894 |
| **Psychiatric conditions** | **ICD-10 codes** |
| Bipolar disorder | F30-F31 |
| Depression/mood disorder | F32-F34, F38-F39, and F41.2 |
| Anxiety | F40-F41 |
| Sleep disorder | F51 and G47 |
| Non-affective psychosis | F20-F29 |
| Stress-related disorder | F42-F48 |
| Eating disorder | F50 |
| Personality disorder | F60-F63 and F68-F69 |
| **Maternal conditions** | **ICD-10 codes** |
| Epilepsy/seizures | G40-G41 |
| Headache/migraine | G43-G44 and R51 |
| Diabetes | E10-E14 |
| Hypertension | I10-I15 and O10-O16 |
| Renal disease | E112, E132, E142, I12-I13, N00-N08, N17-N19, and N25-N27 |
| Gastrointestinal diseases | K20-K21, K25-K29, and K58 |
| Alcohol or drug dependence | F10-F16, F18-F19, Z71.4, Z71.5, Z72.1, and Z72.2 |
| Tobacco dependence | F17, Z71.6, and Z72.0 |
| **Obstetric conditions** | **HIRA procedure codes** |
| Nulliparous | R3131, R3133, R3141, R3143, R4351, R4353, R4361, R4517, R4519, R4507, R4509, R5001, RA361, RA311, RA312, RA315, RA316, RA431, and RA432 |
| Multifetal pregnancy | R3133, R3138, R3143, R3148, R4353, R4358, R4516, R4519, R4520, R5001, R5002, RA312, RA314, RA316, RA318, RA432, and RA434 |
| **Concomitant medications** | **ATC codes** |
| Antidepressants | N06A |
| Anxiolytics | N05B (excl. N05BA) |
| Hypnotics | N05CF |
| Barbiturates | N03AA, N01AF, and N01AG |
| Anticonvulsants | N03A (excl. N03AA and N03AE) |
| Antipsychotics | N05A |
| Stimulants | N06BA |
| Opioid analgesics | N02A |
| Non-insulin antidiabetic agents | A10B |
| Insulin | A10A |
| Antihypertensives | C03A-E, C07, C08C-D, and C09A-D |
| Non-steroidal anti-inflammatory drugs | M01A |
| Triptans | N02CC |
| Lipid-lowering drug | C10 |
| Anti-thyroid drugs | H03B |
| Thyroid hormones | H03AA |
| Systemic corticosteroids | H02AB |
| Azoles | J02AC and D01AC |
| Fertility drugs | G03G |

**Abbreviations:** ATC, Anatomical Therapeutic Chemical Classification; ICD-10, International Classification of Diseases 10^th^ revision; HIRA, Health Insurance Review and Assessment Service of South Korea.
