# Supplementary material for: First-trimester exposure to benzodiazepines and risk of congenital malformations in offspring: A population-based cohort study in South Korea
Source: PLoS Med. 2022 Mar 2;19(3):e1003945. doi: 10.1371/journal.pmed.1003945 (PMC8926183; doi:10.1371/journal.pmed.1003945)
Supplement: S2 Table — (DOCX) [file pmed.1003945.s003.docx]

**S2 Table.** Selection of the study cohort using the HIRA database between 1 July 2009 and 31 December 2019

| **Step** | **Definitions** | **No. of Pregnancies Included** | **No. of Pregnancies Excluded** |
| --- | --- | --- | --- |
| 1 | Pregnancies with live births, 2011-2018 | 3,287,441 | − |
| 2 | Pregnancies linked to liveborn infants | 3,203,306 | 84,135 |
| 3 | Restrict to age 20-45 years | 3,191,341 | 11,965 |
| 4 | Exclude pregnancies with chromosomal abnormality, teratogenic/genetic syndromes, or microdeletions | 3,183,749 | 7,592 |
| 5 | Exclude pregnancies with exposure to known teratogenic drugs* during the first trimester | 3,181,551 | 2,198 |
| 6 | Exclude pregnancies with no BZD prescription during the first trimester but with at least one BZD prescription during the three months before the LMP | 3,094,227 | 87,324 |
| 7 | Identify the study cohort: |  |  |
|  | **Exposed to BZDs:** defined as at least one BZD prescription during the first trimester | 40,846 | − |
|  | **Unexposed to BZDs:** defined as no BZD prescription during the three months before the LMP through the end of the first trimester | 3,053,381 | − |

**Abbreviations:** BZD, benzodiazepines; LMP, last menstrual period; HIRA, Health Insurance Review and Assessment Service

*Known teratogenic drugs: antineoplastic agent, warfarin, lithium, systemic retinoids, misoprostol, thalidomide, androgens, and tetracycline derivative.

With the incidence of congenital malformation = 0.05, exposed:unexposed ratio = 1:50, α = 0.05, and β = 0.2, the total sample size of 4,634 exposed is required to detect a relative risk of 1.2. Therefore, our study was sufficiently powered to detect a potentially increased risk.
