# Supplementary material for: First-trimester exposure to benzodiazepines and risk of congenital malformations in offspring: A population-based cohort study in South Korea
Source: PLoS Med. 2022 Mar 2;19(3):e1003945. doi: 10.1371/journal.pmed.1003945 (PMC8926183; doi:10.1371/journal.pmed.1003945)
Supplement: S1 Fig — (DOCX) [file pmed.1003945.s004.docx]

S1 Fig. Frequency of pregnancies exposed to benzodiazepines during the first trimester in South Korea between 2011 and 2018

|  |  |  | **Year** | | | | | | | | | | | | | | | |
| --- | --- | --- | --- | --- | --- | --- | --- | --- | --- | --- | --- | --- | --- | --- | --- | --- | --- | --- |
|  | **Total** | | **2011** | | **2012** | | **2013** | | **2014** | | **2015** | | **2016** | | **2017** | | **2018** | |
|  | **n** | **(%)** | **n** | **(%)** | **n** | **(%)** | **n** | **(%)** | **n** | **(%)** | **n** | **(%)** | **n** | **(%)** | **n** | **(%)** | **n** | **(%)** |
| **Total no. of pregnancies** | **3,094,227** | | **430,517** | | **443,293** | | **399,193** | | **401,371** | | **405,052** | | **378,100** | | **333,019** | | **303,682** | |
| **Pregnancies exposed to:** |  |  |  |  |  |  |  |  |  |  |  |  |  |  |  |  |  |  |
| **Any benzodiazepines** | **40,846** | **(1.32)** | **6,420** | **(1.49)** | **6,178** | **(1.39)** | **5,463** | **(1.37)** | **4,990** | **(1.24)** | **4,695** | **(1.16)** | **4,128** | **(1.09)** | **3,959** | **(1.19)** | **5,013** | **(1.65)** |
| **Short-acting** |  |  |  |  |  |  |  |  |  |  |  |  |  |  |  |  |  |  |
| Midazolam | 8,609 | (0.28) | 1,084 | (0.25) | 1,123 | (0.25) | 989 | (0.25) | 912 | (0.23) | 843 | (0.21) | 735 | (0.19) | 808 | (0.24) | 2,115 | (0.70) |
| Etizolam | 4,804 | (0.16) | 891 | (0.21) | 836 | (0.19) | 726 | (0.18) | 596 | (0.15) | 528 | (0.13) | 450 | (0.12) | 433 | (0.13) | 344 | (0.11) |
| Lorazepam | 3,665 | (0.12) | 561 | (0.13) | 611 | (0.14) | 539 | (0.14) | 456 | (0.11) | 418 | (0.10) | 411 | (0.11) | 370 | (0.11) | 299 | (0.10) |
| Clotiazepam | 2,468 | (0.08) | 269 | (0.06) | 287 | (0.06) | 311 | (0.08) | 303 | (0.08) | 346 | (0.09) | 296 | (0.08) | 331 | (0.10) | 325 | (0.11) |
| Alprazolam | 1,896 | (0.06) | 360 | (0.08) | 322 | (0.07) | 288 | (0.07) | 230 | (0.06) | 196 | (0.05) | 177 | (0.05) | 190 | (0.06) | 133 | (0.04) |
| Triazolam | 1,473 | (0.05) | 180 | (0.04) | 177 | (0.04) | 186 | (0.05) | 181 | (0.05) | 186 | (0.05) | 169 | (0.04) | 204 | (0.06) | 190 | (0.06) |
| Mexazolam | 1,299 | (0.04) | 171 | (0.04) | 187 | (0.04) | 180 | (0.05) | 168 | (0.04) | 177 | (0.04) | 144 | (0.04) | 148 | (0.04) | 124 | (0.04) |
| **Long-acting** | 56 | (0.00) | 14 | (0.00) | 10 | (0.00) | 7 | (0.00) | 8 | (0.00) | 10 | (0.00) | 1 | (0.00) | 5 | (0.00) | 1 | (0.00) |
| Diazepam |  |  |  |  |  |  |  |  |  |  |  |  |  |  |  |  |  |  |
| Tofisopam | 17,923 | (0.58) | 3,080 | (0.72) | 2,855 | (0.64) | 2,470 | (0.62) | 2,281 | (0.57) | 2,069 | (0.51) | 1,795 | (0.47) | 1,568 | (0.47) | 1,805 | (0.59) |
| Clonazepam | 1,454 | (0.05) | 175 | (0.04) | 118 | (0.03) | 132 | (0.03) | 185 | (0.05) | 192 | (0.05) | 196 | (0.05) | 202 | (0.06) | 254 | (0.08) |
| Flunitrazepam | 824 | (0.03) | 81 | (0.02) | 94 | (0.02) | 85 | (0.02) | 99 | (0.02) | 118 | (0.03) | 108 | (0.03) | 114 | (0.03) | 125 | (0.04) |
| Chlordiazepoxide | 381 | (0.01) | 70 | (0.02) | 62 | (0.01) | 57 | (0.01) | 48 | (0.01) | 48 | (0.01) | 39 | (0.01) | 34 | (0.01) | 23 | (0.01) |
| Clobazam | 218 | (0.01) | 34 | (0.01) | 29 | (0.01) | 26 | (0.01) | 28 | (0.01) | 26 | (0.01) | 23 | (0.01) | 27 | (0.01) | 25 | (0.01) |
| Flurazepam | 159 | (0.01) | 21 | (0.00) | 20 | (0.00) | 21 | (0.01) | 19 | (0.00) | 18 | (0.00) | 19 | (0.01) | 26 | (0.01) | 15 | (0.00) |
| Ethyl loflazepate | 132 | (0.00) | 23 | (0.01) | 29 | (0.01) | 19 | (0.00) | 12 | (0.00) | 15 | (0.00) | 11 | (0.00) | 15 | (0.00) | 8 | (0.00) |
| Clorazepate | 14 | (0.00) | 9 | (0.00) | 1 | (0.00) | 3 | (0.00) | 1 | (0.00) | 0 | (0.00) | 0 | (0.00) | 0 | (0.00) | 0 | (0.00) |
| 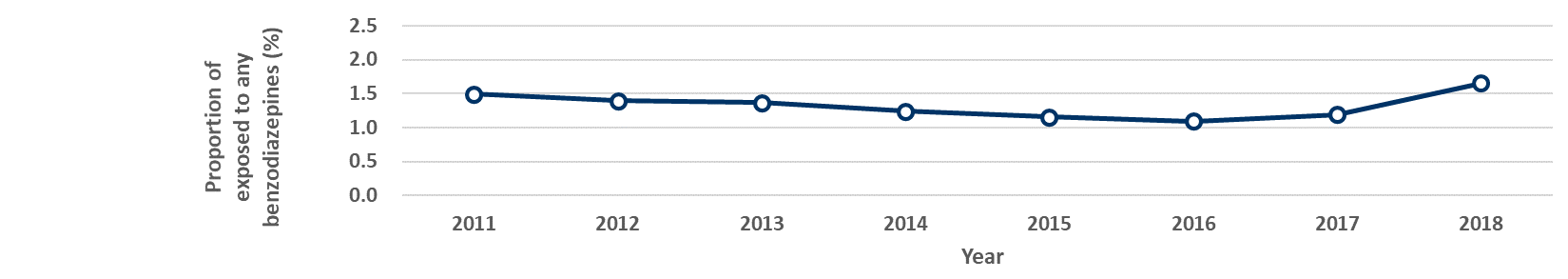 | | | | | | | | | | | | | | | | | | |
