# Supplementary material for: First-trimester exposure to benzodiazepines and risk of congenital malformations in offspring: A population-based cohort study in South Korea
Source: PLoS Med. 2022 Mar 2;19(3):e1003945. doi: 10.1371/journal.pmed.1003945 (PMC8926183; doi:10.1371/journal.pmed.1003945)
Supplement: S2 Fig — (DOCX) [file pmed.1003945.s005.docx]

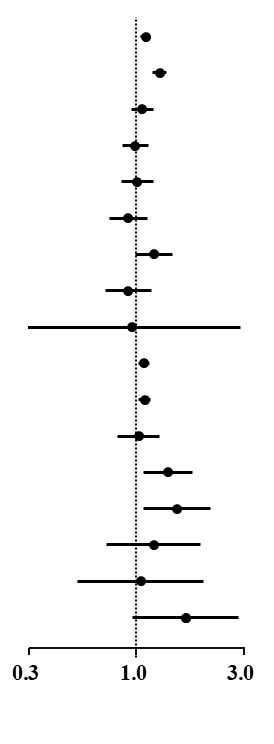
S2 Fig. Absolute and relative risks of overall congenital malformations in infants according to individual benzodiazepine exposure during the first trimester

|  | **No. of  Events** | **No. of  Births** | **Risk/1,000 Births** |  | **Relative Risk (95% CI)** | | **PS-adjusted  relative risk (95% CI)** |
| --- | --- | --- | --- | --- | --- | --- | --- |
| **Subgroups** |  |  |  |  | **Unadjusted** | **PS-adjusted** |  |
| **Overall congenital malformations** |  |  |  |  |  |  |  |
| **Short-acting** | 1,482 | 21,775 | 68.1 |  | 1.32 (1.26–1.39) | **1.09 (1.03–1.14)** |  |
| Midazolam | 741 | 8,609 | 86.1 |  | 1.68 (1.56–1.79) | **1.26 (1.17–1.35)** |  |
| Tofisopam | 274 | 4,804 | 57.0 |  | 1.11 (0.99–1.25) | 1.05 (0.94–1.18) |  |
| Etizolam | 204 | 3,665 | 55.7 |  | 1.08 (0.95–1.24) | 0.98 (0.86–1.12) |  |
| Lorazepam | 154 | 2,468 | 62.4 |  | 1.21 (1.04–1.42) | 1.00 (0.85–1.18) |  |
| Clotiazepam | 95 | 1,896 | 50.1 |  | 0.98 (0.80–1.19) | 0.91 (0.75–1.11) |  |
| Alprazolam | 105 | 1,473 | 71.3 |  | 1.39 (1.15–1.67) | 1.19 (0.98–1.44) |  |
| Triazolam | 70 | 1,299 | 53.9 |  | 1.05 (0.83–1.32) | 0.91 (0.72–1.16) |  |
| Mexazolam | 3 | 56 | 53.6 |  | 1.04 (0.35–3.13) | 0.95 (0.32–2.86) |  |
| **Long-acting** | 1,271 | 20,321 | 62.5 |  | 1.22 (1.15–1.28) | **1.07 (1.01–1.13)** |  |
| Diazepam | 1,110 | 17,923 | 61.9 |  | 1.21 (1.14–1.28) | **1.08 (1.01–1.14)** |  |
| Clonazepam | 93 | 1,454 | 64.0 |  | 1.24 (1.02–1.52) | 1.02 (0.82–1.25) |  |
| Flunitrazepam | 68 | 824 | 82.5 |  | 1.61 (1.28–2.02) | **1.37 (1.07–1.76)** |  |
| Chlordiazepoxide | 30 | 381 | 78.7 |  | 1.53 (1.09–2.16) | **1.50 (1.06–2.11)** |  |
| Clobazam | 17 | 218 | 78.0 |  | 1.52 (0.96–2.40) | 1.18 (0.73–1.91) |  |
| Flurazepam | 9 | 159 | 56.6 |  | 1.10 (0.58–2.08) | 1.04 (0.54–1.98) |  |
| Ethyl loflazepate | 12 | 132 | 90.9 |  | 1.77 (1.03–3.03) | 1.64 (0.96–2.82) |  |
|  |  |  |  |  |  |  |  |
|  |  |  |  |  |  |  |  |

**Abbreviations:** PS, propensity score; CI, confidence interval.

Clorazepate was not analysed due to the small sample size (n=14).
