# Supplementary material for: First-trimester exposure to benzodiazepines and risk of congenital malformations in offspring: A population-based cohort study in South Korea
Source: PLoS Med. 2022 Mar 2;19(3):e1003945. doi: 10.1371/journal.pmed.1003945 (PMC8926183; doi:10.1371/journal.pmed.1003945)
Supplement: S3 Fig — (DOCX) [file pmed.1003945.s006.docx]

S3 Fig. Absolute and relative risks of congenital heart defects in infants according to individual benzodiazepine exposure during the first trimester

|  | **No. of  Events** | **No. of  Births** | **Risk/1,000 Births** |  | **Relative Risk (95% CI)** | | **PS-adjusted  relative risk (95% CI)** |
| --- | --- | --- | --- | --- | --- | --- | --- |
| **Subgroups** |  |  |  |  | **Unadjusted** | **PS-adjusted** |  |
| **Congenital heart defects** |  |  |  |  |  |  |  |
| **Short-acting** | 913 | 21,775 | 41.9 |  | 1.53 (1.44–1.63) | **1.18 (1.10–1.26)** |  |
| Midazolam | 470 | 8,609 | 54.6 |  | 1.99 (1.83–2.18) | **1.34 (1.22–1.47)** |  |
| Tofisopam | 153 | 4,804 | 31.8 |  | 1.16 (1.00–1.36) | 1.09 (0.93–1.28) |  |
| Etizolam | 133 | 3,665 | 36.3 |  | 1.33 (1.12–1.57) | 1.16 (0.98–1.38) |  |
| Lorazepam | 98 | 2,468 | 39.7 |  | 1.45 (1.19–1.76) | 1.14 (0.93–1.40) |  |
| Clotiazepam | 51 | 1,896 | 26.9 |  | 0.98 (0.75–1.29) | 0.90 (0.69–1.19) |  |
| Alprazolam | 65 | 1,473 | 44.1 |  | 1.61 (1.27–2.04) | **1.33 (1.04–1.71)** |  |
| Triazolam | 45 | 1,299 | 34.6 |  | 1.27 (0.95–1.69) | 1.12 (0.83–1.51) |  |
| Mexazolam | 1 | 56 | 17.9 |  | 0.65 (0.09–4.55) | 0.57 (0.08–3.98) |  |
| **Long–acting** | 733 | 20,321 | 36.1 |  | 1.32 (1.23–1.42) | **1.11 (1.04–1.20)** | 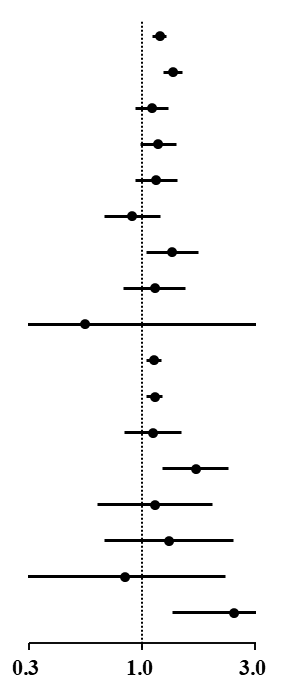 |
| Diazepam | 641 | 17,923 | 35.8 |  | 1.31 (1.21–1.41) | **1.12 (1.04–1.21)** |  |
| Clonazepam | 58 | 1,454 | 39.9 |  | 1.46 (1.13–1.88) | 1.10 (0.84–1.45) |  |
| Flunitrazepam | 43 | 824 | 52.2 |  | 1.91 (1.42–2.55) | **1.67 (1.21–2.29)** |  |
| Chlordiazepoxide | 12 | 381 | 31.5 |  | 1.15 (0.66–2.01) | 1.13 (0.65–1.97) |  |
| Clobazam | 11 | 218 | 50.5 |  | 1.84 (1.04–3.28) | 1.28 (0.69–2.40) |  |
| Flurazepam | 4 | 159 | 25.2 |  | 0.92 (0.35–2.42) | 0.84 (0.31–2.23) |  |
| Ethyl loflazepate | 10 | 132 | 75.8 |  | 2.77 (1.53–5.02) | **2.43 (1.33–4.42)** |  |
|  |  |  |  |  |  |  |  |
|  |  |  |  |  |  |  |  |

**Abbreviations:** PS, propensity score; CI, confidence interval.

Clorazepate was not analysed due to the small sample size (n=14).
