# Supplementary material for: First-trimester exposure to benzodiazepines and risk of congenital malformations in offspring: A population-based cohort study in South Korea
Source: PLoS Med. 2022 Mar 2;19(3):e1003945. doi: 10.1371/journal.pmed.1003945 (PMC8926183; doi:10.1371/journal.pmed.1003945)
Supplement: S4 Fig — (DOCX) [file pmed.1003945.s007.docx]

S4 Fig. Risks of congenital malformations in infants following maternal exposure to benzodiazepines during the first trimester: sensitivity analyses

|  | **Benzodiazepine** | |  | **Unexposed** | |  | **Relative Risk (95% CI)** | | **PS-adjusted  relative risk (95% CI)** |
| --- | --- | --- | --- | --- | --- | --- | --- | --- | --- |
|  | **No. of  Events** | **No. of**  **Births** |  | **No. of  Events** | **No. of**  **Births** |  | **Unadjusted** | **PS-adjusted** |  |
| **Nervous system** |  |  |  |  |  |  |  |  |  |
| Main analysis | 155 | 40,846 |  | 10,032 | 3,053,381 |  | 1.15 (0.99–1.35) | 1.05 (0.89–1.24) |  |
| ≥2 prescriptions of exposure | 104 | 27,809 |  | 10,032 | 3,053,381 |  | 1.14 (0.94–1.38) | 1.09 (0.90–1.34) |  |
| ≥2 diagnoses of outcome | 80 | 40,846 |  | 5,031 | 3,053,381 |  | 1.19 (0.95–1.48) | 1.05 (0.84–1.33) |  |
| Restriction to women with main indications | 47 | 11,603 |  | 265 | 74,129 |  | 1.13 (0.83–1.54) | 1.13 (0.81–1.57) |  |
| Restriction to nulliparous women | 89 | 20,477 |  | 5,795 | 1,601,759 |  | 1.20 (0.97–1.48) | 1.04 (0.84–1.29) |  |
| Negative control analysis | 385 | 105,912 |  | 9,555 | 2,922,168 |  | 1.11 (1.00–1.23) | 1.06 (0.95–1.18) |  |
| **Eye** |  |  |  |  |  |  |  |  |  |
| Main analysis | 57 | 40,846 |  | 4,044 | 3,053,381 |  | 1.05 (0.81–1.37) | 0.99 (0.75–1.29) |  |
| ≥2 prescriptions of exposure | 32 | 27,809 |  | 4,044 | 3,053,381 |  | 0.87 (0.61–1.23) | 0.83 (0.58–1.19) |  |
| ≥2 diagnoses of outcome | 41 | 40,846 |  | 2,691 | 3,053,381 |  | 1.14 (0.84–1.55) | 1.03 (0.75–1.43) |  |
| Restriction to women with main indications | 13 | 11,603 |  | 87 | 74,129 |  | 0.95 (0.53–1.71) | 0.92 (0.49–1.72) |  |
| Restriction to nulliparous women | 29 | 20,477 |  | 2,067 | 1,601,759 |  | 1.10 (0.76–1.58) | 1.02 (0.70–1.50) |  |
| Negative control analysis | 141 | 105,912 |  | 3,864 | 2,922,168 |  | 1.01 (0.85–1.19) | 0.95 (0.80–1.13) |  |
| **Ear, face, and neck** |  |  |  |  |  |  |  |  |  |
| Main analysis | 27 | 40,846 |  | 1,835 | 3,053,381 |  | 1.10 (0.75–1.61) | 1.04 (0.70–1.53) |  |
| ≥2 prescriptions of exposure | 13 | 27,809 |  | 1,835 | 3,053,381 |  | 0.78 (0.45–1.34) | 0.76 (0.44–1.31) |  |
| ≥2 diagnoses of outcome | 11 | 40,846 |  | 1,035 | 3,053,381 |  | 0.79 (0.44–1.44) | 0.76 (0.42–1.38) |  |
| Restriction to women with main indications | 7 | 11,603 |  | 46 | 74,129 |  | 0.97 (0.44–2.15) | 1.05 (0.46–2.37) | 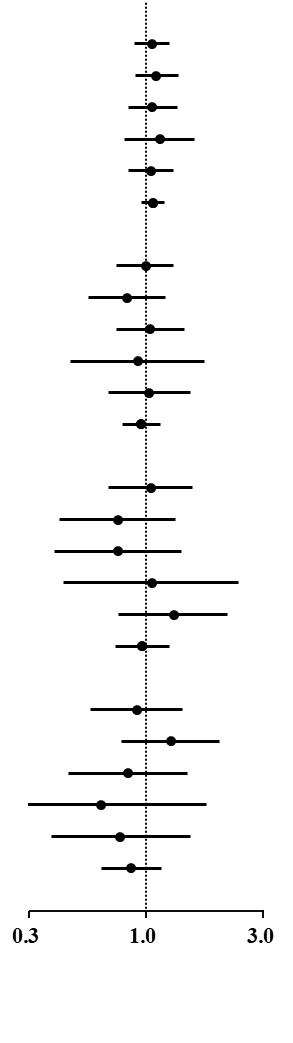 |
| Restriction to nulliparous women | 16 | 20,477 |  | 887 | 1,601,759 |  | 1.41 (0.86–2.31) | 1.29 (0.77–2.14) |  |
| Negative control analysis | 67 | 105,912 |  | 1,749 | 2,922,168 |  | 1.06 (0.83–1.35) | 0.96 (0.74–1.24) |  |
| **Respiratory system** |  |  |  |  |  |  |  |  |  |
| Main analysis | 23 | 40,846 |  | 1,658 | 3,053,381 |  | 1.04 (0.69–1.56) | 0.91 (0.59–1.40) |  |
| ≥2 prescriptions of exposure | 20 | 27,809 |  | 1,658 | 3,053,381 |  | 1.32 (0.85–2.06) | 1.25 (0.79–1.97) |  |
| ≥2 diagnoses of outcome | 13 | 40,846 |  | 1,139 | 3,053,381 |  | 0.85 (0.49–1.47) | 0.84 (0.48–1.47) |  |
| Restriction to women with main indications | 5 | 11,603 |  | 40 | 74,129 |  | 0.80 (0.32–2.02) | 0.65 (0.24–1.75) |  |
| Restriction to nulliparous women | 10 | 20,477 |  | 869 | 1,601,759 |  | 0.90 (0.48–1.68) | 0.78 (0.41–1.50) |  |
| Negative control analysis | 57 | 105,912 |  | 1,587 | 2,922,168 |  | 0.99 (0.76–1.29) | 0.87 (0.65–1.15) |  |
|  |  |  |  |  |  |  |  |  |  |
|  |  |  |  |  |  |  |  |  |  |

**Abbreviations:** PS, propensity score; CI, confidence interval.
