# Supplementary material for: First-trimester exposure to benzodiazepines and risk of congenital malformations in offspring: A population-based cohort study in South Korea
Source: PLoS Med. 2022 Mar 2;19(3):e1003945. doi: 10.1371/journal.pmed.1003945 (PMC8926183; doi:10.1371/journal.pmed.1003945)
Supplement: S5 Fig — (DOCX) [file pmed.1003945.s008.docx]

S5 Fig. Risks of congenital malformations in infants following maternal exposure to benzodiazepines during the first trimester: sensitivity analyses Ⅱ

|  | **Benzodiazepine** | |  | **Unexposed** | |  | **Relative Risk (95% CI)** | | **PS-adjusted  relative risk (95% CI)** |
| --- | --- | --- | --- | --- | --- | --- | --- | --- | --- |
|  | **No. of  Events** | **No. of**  **Births** |  | **No. of  Events** | **No. of**  **Births** |  | **Unadjusted** | **PS-adjusted** |  |
| **Oral clefts** |  |  |  |  |  |  |  |  |  |
| Main analysis | 60 | 40,846 |  | 4,252 | 3,053,381 |  | 1.05 (0.82–1.36) | 0.89 (0.68–1.16) |  |
| ≥2 prescriptions of exposure | 38 | 27,809 |  | 4,252 | 3,053,381 |  | 0.98 (0.71–1.35) | 0.84 (0.60–1.17) |  |
| ≥2 diagnoses of outcome | 51 | 40,846 |  | 3,343 | 3,053,381 |  | 1.14 (0.87–1.50) | 0.99 (0.74–1.32) |  |
| Restriction to women with main indications | 22 | 11,603 |  | 127 | 74,129 |  | 1.11 (0.70–1.74) | 1.09 (0.67–1.75) |  |
| Restriction to nulliparous women | 26 | 20,477 |  | 2,127 | 1,601,759 |  | 0.96 (0.65–1.41) | 0.76 (0.50–1.13) |  |
| Negative control analysis | 169 | 105,912 |  | 4,039 | 2,922,168 |  | 1.15 (0.99–1.35) | 1.01 (0.86–1.20) |  |
| **Digestive system** |  |  |  |  |  |  |  |  |  |
| Main analysis | 142 | 40,846 |  | 8,697 | 3,053,381 |  | 1.22 (1.03–1.44) | 1.13 (0.95–1.35) |  |
| ≥2 prescriptions of exposure | 97 | 27,809 |  | 8,697 | 3,053,381 |  | 1.22 (1.00–1.50) | 1.16 (0.94–1.42) |  |
| ≥2 diagnoses of outcome | 91 | 40,846 |  | 5,043 | 3,053,381 |  | 1.35 (1.10–1.66) | 1.23 (0.99–1.53) |  |
| Restriction to women with main indications | 40 | 11,603 |  | 232 | 74,129 |  | 1.10 (0.79–1.54) | 1.12 (0.78–1.60) |  |
| Restriction to nulliparous women | 70 | 20,477 |  | 4,511 | 1,601,759 |  | 1.21 (0.96–1.54) | 1.09 (0.85–1.39) | 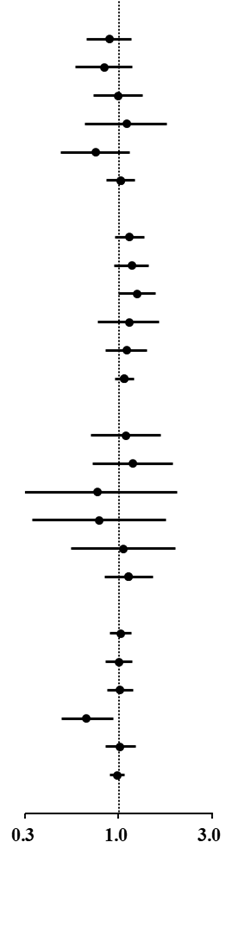 |
| Negative control analysis | 344 | 105,912 |  | 8,283 | 2,922,168 |  | 1.15 (1.03–1.28) | 1.06 (0.95–1.19) |  |
| **Abdominal wall** |  |  |  |  |  |  |  |  |  |
| Main analysis | 27 | 40,846 |  | 1,284 | 3,053,381 |  | 1.57 (1.07–2.30) | 1.08 (0.72–1.63) |  |
| ≥2 prescriptions of exposure | 20 | 27,809 |  | 1,284 | 3,053,381 |  | 1.71 (1.10–2.66) | 1.17 (0.73–1.88) |  |
| ≥2 diagnoses of outcome | 5 | 40,846 |  | 291 | 3,053,381 |  | 1.28 (0.53–3.11) | 0.77 (0.30–1.97) |  |
| Restriction to women with main indications | 8 | 11,603 |  | 51 | 74,129 |  | 1.00 (0.48–2.11) | 0.79 (0.36–1.74) |  |
| Restriction to nulliparous women | 12 | 20,477 |  | 653 | 1,601,759 |  | 1.44 (0.81–2.54) | 1.05 (0.57–1.94) |  |
| Negative control analysis | 60 | 105,912 |  | 1,212 | 2,922,168 |  | 1.37 (1.05–1.77) | 1.12 (0.84–1.49) |  |
| **Urinary system** |  |  |  |  |  |  |  |  |  |
| Main analysis | 258 | 40,846 |  | 16,876 | 3,053,381 |  | 1.14 (1.01–1.29) | 1.02 (0.90–1.16) |  |
| ≥2 prescriptions of exposure | 165 | 27,809 |  | 16,876 | 3,053,381 |  | 1.07 (0.92–1.25) | 1.00 (0.85–1.17) |  |
| ≥2 diagnoses of outcome | 180 | 40,846 |  | 11,454 | 3,053,381 |  | 1.17 (1.01–1.36) | 1.01 (0.87–1.18) |  |
| Restriction to women with main indications | 51 | 11,603 |  | 438 | 74,129 |  | 0.74 (0.56–0.99) | 0.68 (0.51–0.93) |  |
| Restriction to nulliparous women | 135 | 20,477 |  | 8,963 | 1,601,759 |  | 1.18 (0.99–1.40) | 1.01 (0.85–1.21) |  |
| Negative control analysis | 613 | 105,912 |  | 16,104 | 2,922,168 |  | 1.05 (0.97–1.14) | 0.98 (0.90–1.07) |  |
|  |  |  |  |  |  |  |  |  |  |
|  |  |  |  |  |  |  |  |  |  |

**Abbreviations:** PS, propensity score; CI, confidence interval.
