# Supplementary material for: First-trimester exposure to benzodiazepines and risk of congenital malformations in offspring: A population-based cohort study in South Korea
Source: PLoS Med. 2022 Mar 2;19(3):e1003945. doi: 10.1371/journal.pmed.1003945 (PMC8926183; doi:10.1371/journal.pmed.1003945)
Supplement: S6 Fig — (DOCX) [file pmed.1003945.s009.docx]

S6 Fig. Risks of congenital malformations in infants following maternal exposure to benzodiazepines during the first trimester: sensitivity analyses III

|  | **Benzodiazepine** | |  | **Unexposed** | |  | **Relative Risk (95% CI)** | | **PS-adjusted  relative risk (95% CI)** |
| --- | --- | --- | --- | --- | --- | --- | --- | --- | --- |
|  | **No. of  Events** | **No. of**  **Births** |  | **No. of  Events** | **No. of**  **Births** |  | **Unadjusted** | **PS-adjusted** |  |
| **Genital organs** |  |  |  |  |  |  |  |  |  |
| Main analysis | 227 | 40,846 |  | 13,514 | 3,053,381 |  | 1.26 (1.10–1.43) | 1.11 (0.97–1.27) |  |
| ≥2 prescriptions of exposure | 142 | 27,809 |  | 13,514 | 3,053,381 |  | 1.15 (0.98–1.36) | 1.07 (0.90–1.27) |  |
| ≥2 diagnoses of outcome | 122 | 40,846 |  | 6,960 | 3,053,381 |  | 1.31 (1.10–1.57) | 1.11 (0.92–1.34) |  |
| Restriction to women with main indications | 60 | 11,603 |  | 343 | 74,129 |  | 1.12 (0.85–1.47) | 1.11 (0.83–1.49) |  |
| Restriction to nulliparous women | 124 | 20,477 |  | 7,054 | 1,601,759 |  | 1.38 (1.15–1.64) | 1.17 (0.97–1.41) |  |
| Negative control analysis | 528 | 105,912 |  | 12,861 | 2,922,168 |  | 1.13 (1.04–1.24) | 1.00 (0.91–1.10) |  |
| **Limb** |  |  |  |  |  |  |  |  |  |
| Main analysis | 184 | 40,846 |  | 12,956 | 3,053,381 |  | 1.06 (0.92–1.23) | 0.94 (0.80–1.09) |  |
| ≥2 prescriptions of exposure | 122 | 27,809 |  | 12,956 | 3,053,381 |  | 1.03 (0.87–1.24) | 0.92 (0.77–1.11) |  |
| ≥2 diagnoses of outcome | 71 | 40,846 |  | 5,650 | 3,053,381 |  | 0.94 (0.74–1.19) | 0.83 (0.65–1.06) |  |
| Restriction to women with main indications | 52 | 11,603 |  | 363 | 74,129 |  | 0.92 (0.68–1.22) | 0.85 (0.63–1.15) |  |
| Restriction to nulliparous women | 113 | 20,477 |  | 6,937 | 1,601,759 |  | 1.27 (1.06–1.53) | 1.10 (0.90–1.33) |  |
| Negative control analysis | 504 | 105,912 |  | 12,326 | 2,922,168 |  | 1.13 (1.03–1.23) | 0.97 (0.88–1.07) |  |
| **Others** |  |  |  |  |  |  |  |  |  |
| Main analysis | 149 | 40,846 |  | 8,927 | 3,053,381 |  | 1.25 (1.06–1.47) | 1.13 (0.95–1.34) |  |
| ≥2 prescriptions of exposure | 89 | 27,809 |  | 8,927 | 3,053,381 |  | 1.09 (0.89–1.35) | 1.04 (0.84–1.29) |  |
| ≥2 diagnoses of outcome | 67 | 40,846 |  | 3,892 | 3,053,381 |  | 1.29 (1.01–1.64) | 1.12 (0.87–1.44) |  |
| Restriction to women with main indications | 39 | 11,603 |  | 228 | 74,129 |  | 1.09 (0.78–1.53) | 1.09 (0.76–1.56) | 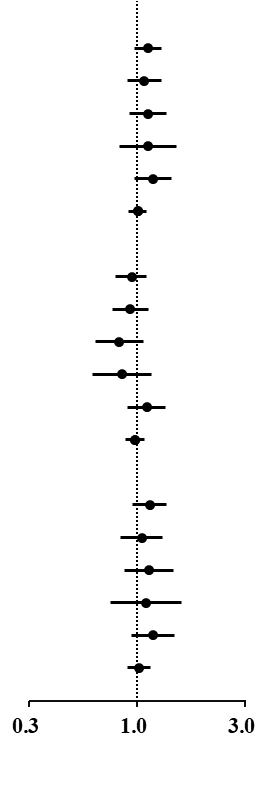 |
| Restriction to nulliparous women | 88 | 20,477 |  | 4,956 | 1,601,759 |  | 1.39 (1.13–1.71) | 1.17 (0.94–1.46) |  |
| Negative control analysis | 334 | 105,912 |  | 8,489 | 2,922,168 |  | 1.09 (0.97–1.21) | 1.01 (0.90–1.14) |  |
|  |  |  |  |  |  |  |  |  |  |
|  |  |  |  |  |  |  |  |  |  |

**Abbreviations:** PS, propensity score; CI, confidence interval.
