# Supplementary material for: First-trimester exposure to benzodiazepines and risk of congenital malformations in offspring: A population-based cohort study in South Korea
Source: PLoS Med. 2022 Mar 2;19(3):e1003945. doi: 10.1371/journal.pmed.1003945 (PMC8926183; doi:10.1371/journal.pmed.1003945)
Supplement: S1 Appendix — (DOCX) [file pmed.1003945.s010.docx]

S1 Appendix. Sensitivity analysis of unmeasured confounders (Rule-out approach)

As maternal smoking status is not captured in our claims data, residual confounding is of concern in terms of the increased risk of congenital malformations and congenital heart defects. Thus, we quantified the potential impact of unmeasured confounders (e.g., tobacco use) using the rule-out approach.^1^ This approach evaluates the following question: How strongly does an unmeasured confounder have to fully explain the observed findings?

According to the Korea National Health and Nutrition Examination Survey, approximately 6.5% of women smoke cigarettes during pregnancy.^2^ Figures in the next page show the extent of association between exposure (benzodiazepine exposure during the first trimester) and a confounder, noted as OR_EC_, and the association between confounders and outcomes (overall congenital malformations and congenital heart defects), noted as RR_CD_, which are necessary to fully explain the elevated observed risk (congenital malformations overall:1.09, congenital heart defects:1.15).

If the confounder exists in 6.5% of the population, a relative risk of 2 or more for both OR_EC_ and RR_CD_ is needed to fully explain the observed findings. On hypothesising the prevalence of confounders as 20% of the population, relative risks of approximately 1.8 or more are required. However, a previous study has reported the OR for smoking and overall congenital malformations and heart defects as 1.01 and 1.09, respectively,^3^ which indicates that our result is unlikely to be due to unmeasured confounders.

Note: Each line splits the area into two parts. The upper right area represents all combinations of an association between exposure and confounder (OR_EC_) and an association between confounder and outcome (RR_CD_) that would create confounding by an unmeasured factor strong enough to move the point estimate of RR to the null value (RR=1) or beyond. The lower-left area represents all parameter combinations that would not move the estimated RR toward the null.

**References**

1. Schneeweiss S. Sensitivity analysis and external adjustment for unmeasured confounders in epidemiologic database studies of therapeutics. Pharmacoepidemiology and drug safety 2006;15(5):291-303.

2. Joo HS, Kim C-B, Nam EW, et al. Comparison of Health-related Behaviors in Pregnant Women and Breast-feeding Mothers vs Non-pregnant Women. Korean J Women Health Nurs 2014;20(3):185-94.

3. Hackshaw A, Rodeck C, Boniface S. Maternal smoking in pregnancy and birth defects: a systematic review based on 173 687 malformed cases and 11.7 million controls. Human reproduction update 2011;17(5):589-604.

Fig. Sensitivity analysis of unmeasured confounders (prevalence of confounder [Pc]: 6.5%, 20%) for the estimated adjusted relative risk of (a) 1.09 for overall congenital malformations and (b) 1.15 for congenital heart defects

(a)

(b)
