# Supplementary material for: First-trimester exposure to benzodiazepines and risk of congenital malformations in offspring: A population-based cohort study in South Korea
Source: PLoS Med. 2022 Mar 2;19(3):e1003945. doi: 10.1371/journal.pmed.1003945 (PMC8926183; doi:10.1371/journal.pmed.1003945)
Supplement: S2 Appendix — (DOCX) [file pmed.1003945.s011.docx]

S2 Appendix. Potential effect of including live births only

Our study included pregnancies that resulted in live births only and did not include terminated pregnancies (e.g., abortion and stillbirths). If the probability of live birth differs between benzodiazepine-exposed and unexposed pregnancies, this may introduce selection bias. If the probability of live birth is lower in benzodiazepine-exposed pregnancies than in unexposed pregnancies, the risk estimates may be underestimated. For example, BZD-exposed women could be more likely to have careful screening tests and detect congenital malformations during pregnancy. Also, pregnancy loss could occur more in BZD-exposed women than in unexposed women. Thus, we quantified the potential effects of missing live births.

Considering a difference in probability of live births between benzodiazepine-exposed and unexposed pregnancies, the corrected relative risks were calculated as follows:

Corrected RR=Observed RR*(S_10_*S_01_/S_11_*S_00_)

S_00_ refers to the probability of live births in benzodiazepine-unexposed pregnancies without malformation.
S_01_ refers to the probability of live births in benzodiazepine-exposed pregnancies without malformation.
S_10_ refers to the probability of live births in benzodiazepine-unexposed pregnancies with malformation.
S_11_ refers to the probability of live births in benzodiazepine-exposed pregnancies with malformation.

This method has been widely used in studies that assess the association between medication use during pregnancy and the risk of malformations. Detailed explanations are provided in previous studies.^1,2^

Based on estimates from the literature, the live birth probability among benzodiazepine-unexposed pregnancies without malformation (S_00_) was defined as 80%.^3^ We then assumed the probability of live birth among unexposed pregnancies with malformations (S_10_) as a range of 55% to 80%, based on a previous study.^4^ Lastly, we evaluated the potential effect of lower frequency of live birth, ranging from 10% to 20%, in benzodiazepine-exposed pregnancies. This estimation is based on studies that reported a 1.8-fold higher risk of spontaneous abortion in benzodiazepine-exposed pregnancies than in unexposed pregnancies and nondifferential risk of stillbirth between benzodiazepine-exposed and unexposed pregnancies, as stillbirth occurs in less than 1% of all births and stillbirths due to malformations may be rare.^5-7^

Table. Sensitivity analysis to assess the impact of restriction on live births based on probabilities of live births in pregnancy exposure groups with and without malformations

| **Pregnancies with malformations** | | **Pregnancies without malformations** | |
| --- | --- | --- | --- |
| **Unexposed (S**_10_**)** | **Benzodiazepine exposed (S**_11_**)** | **Unexposed (S**_00_**)** | **Benzodiazepine exposed (S**_01_**)** |
| 55–80% | S_10_-20% | 80% | S_00_-20% |
|  | S_10_-10% |  | S_00_-10% |
|  | S_10_ |  | S_00_ |

Figures below present the corrected RR for overall congenital malformations and heart defects, respectively. Starting from the estimates in our primary analysis (RR = 1.09 and 1.15), the risk remained below 1.4, under the most extreme scenario (assuming the selection probability of benzodiazepine-exposed pregnancies with malformation as 35%), which is highly unlikely.

Fig. Corrected relative risk for the association between benzodiazepine exposure during the first trimester and (a) overall congenital malformations and (b) congenital heart defects

(a) Overall congenital malformations, starting from the observed adjusted relative risk of 1.09


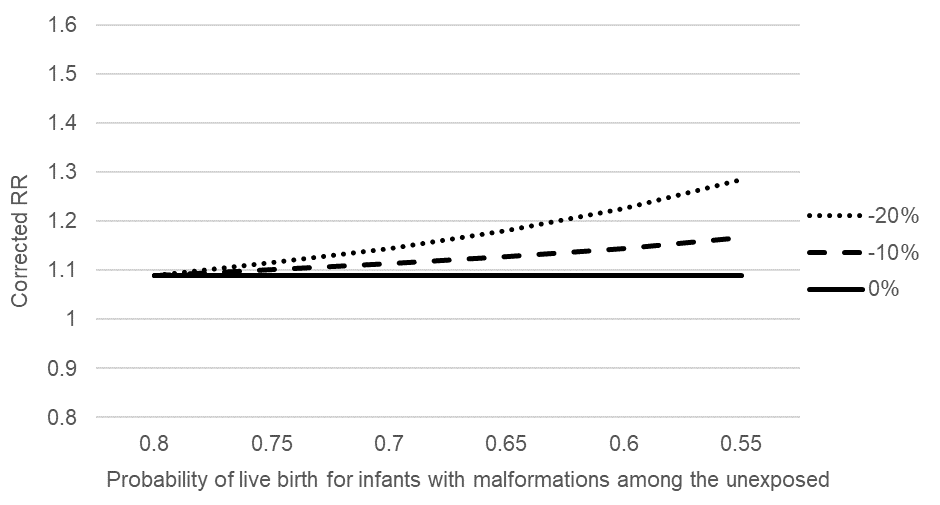


(b) Congenital heart defects, starting from the observed adjusted relative risk of 1.15


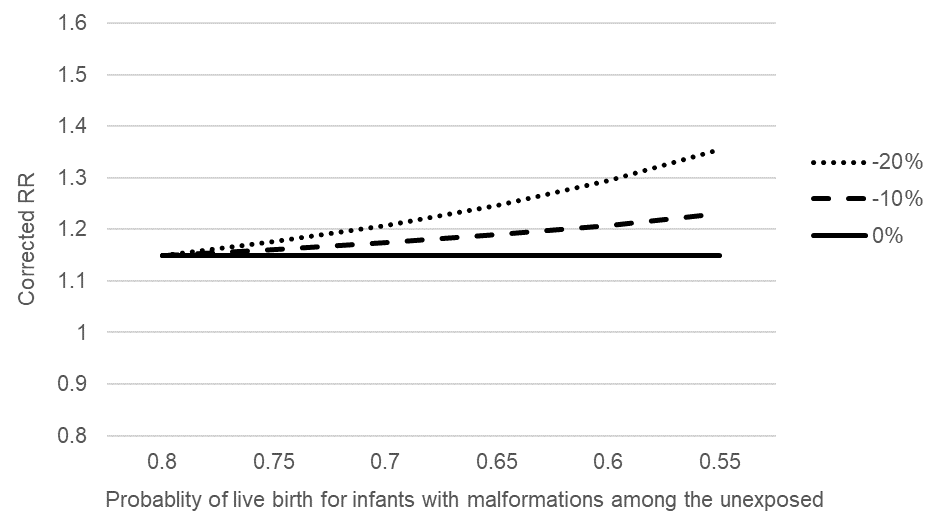


**References**

1. Huybrechts KF, Palmsten K, Avorn J, et al. Antidepressant use in pregnancy and the risk of cardiac defects. New England Journal of Medicine 2014;370(25):2397-407.

2. Patorno E, Huybrechts KF, Bateman BT, et al. Lithium use in pregnancy and the risk of cardiac malformations. New England Journal of Medicine 2017;376(23):2245-54.

3. Lee S-Y. 2018 National Survey on Fertility, Family Health and Welfare: Korea Institute for Health and Social Affairs, 2019.

4. Svensson E, Ehrenstein V, Nørgaard M, et al. Brief Report: Estimating the Proportion of All Observed Birth Defects Occurring in Pregnancies Terminated by a Second-trimester Abortion. Epidemiology 2014:866-71.

5. Sheehy O, Zhao J-P, Bérard A. Association between incident exposure to benzodiazepines in early pregnancy and risk of spontaneous abortion. JAMA psychiatry 2019;76(9):948-57.

6. Say L, Donner A, Gülmezoglu AM, et al. The prevalence of stillbirths: a systematic review. Reprod Health 2006;3:1. doi: 10.1186/1742-4755-3-1 [published Online First: 2006/01/13]

7. Stillbirth Collaborative Research Network Writing Group. Causes of death among stillbirths. Jama 2011;306(22):2459-68. doi: 10.1001/jama.2011.1823 [published Online First: 2011/12/15]
