# Supplementary material for: First-trimester exposure to benzodiazepines and risk of congenital malformations in offspring: A population-based cohort study in South Korea
Source: PLoS Med. 2022 Mar 2;19(3):e1003945. doi: 10.1371/journal.pmed.1003945 (PMC8926183; doi:10.1371/journal.pmed.1003945)
Supplement: S1 Protocol — (DOCX) [file pmed.1003945.s012.docx]

**SUMMARY PROTOCOL**

| **Protocol Title:** | Maternal and Neonatal Outcomes Following Exposure to Benzodiazepines During Pregnancy |
| --- | --- |
| **Study Objective:** | To assess the association between benzodiazepine use in early pregnancy and the risk of congenital malformations |
| **Study type:** | Observational |
| **Study Design:** | A retrospective nationwide cohort study using the Health Insurance Review and Assessment (HIRA) database of South Korea, which covers the entire South Korean population. |
| **Study Population:** | Pregnant women who gave birth in South Korea between 2011 and 2018  **Sex/Gender:** Female  **Ages:** 20 years to 45 years (adult)  **Inclusion Criteria:**   - Pregnancies with live birth, 2011-2018 - Pregnancies linked to liveborn infants - Pregnancies aged 20-45 years at delivery   **Exclusion Criteria:**   - Pregnancies with a chromosomal abnormality - Pregnancies with exposure to known teratogenic drugs (e.g., antineoplastic agent, warfarin, lithium, isotretinoin, misoprostol, thalidomide) during the first trimester - Pregnancies with no benzodiazepine prescription during the first trimester, but with at least one benzodiazepine prescription during the 3 months before the pregnancy onset |
| **Intervention:** | Exposure to benzodiazepine during early pregnancy  **Groups:**  1) Pregnancies with exposure to benzodiazepines:  Women receiving at least one benzodiazepine prescription during the first trimester (first 90 days of pregnancy).  2) Pregnancies without exposure to benzodiazepines:  Women who did not receive a benzodiazepine prescription during the three months before the pregnancy onset through the end of the first trimester |
| **Sample Size:** | Based on the estimated number of live births during 2011-2018 from the Korean Statistical Information Service (KOSIS), we anticipated that our study cohort would include approximately 3 million pregnancies in this study. |
| **Study Endpoints:** | Risk of congenital malformations [Time Frame: from the birth date until up to 8 years, death, or study end date (Dec 31, 2019)]  Overall congenital malformations and organ-specific congenital malformations in infants, which are confirmed by diagnostic records in the HIRA database. All infants were followed up for at least one year. |
| **Statistical Methods:** | **Main analysis:**   - The balance of characteristics between the two groups will be evaluated using an absolute standardized difference (aSD), where a value >0.1 indicates a significant imbalance. - The absolute risks (per 1,000 pregnancies), risk differences, and unadjusted relative risks (RR) with 95% confidence interval (CI) for risk of major malformations were calculated, stratified by exposure to benzodiazepines. - Propensity score (PS) fine stratification method (PS weighting after fine stratification) will be used to control for potential confounders and the surrogates of potential confounders. - The adjusted RR with 95% CI will be estimated using a weighted generalized linear model (log-binomial).   **Subgroup analysis:**   - We will conduct seven pre-specified subgroup analyses as below: - Dose-response relationship - Duration of action (short-acting vs. long-acting) - Individual ingredients - Four maternal characteristics (potential risk factors for congenital malformations): maternal age, multiple pregnancy, epilepsy, and concomitant use of antidepressants   **Sensitivity analysis:**   1. Exposure will redefine as having two or more BZD prescriptions in the first trimester to minimize exposure misclassification. 2. The outcome will redefine as the presence of ≥2 diagnoses of congenital malformations to consider possible outcome misclassification. 3. We will restrict the study cohort to those who had underlying comorbidities related to the primary indication for benzodiazepines (e.g., bipolar disorder, depression/mood disorder, anxiety, sleep disorder, and gastrointestinal disease) to mitigate confounding by indication. 4. We will restrict the study cohort to nulliparous women to account for intra-individual correlations. 5. We will conduct a negative control analysis (NCA) by ascertaining exposure between 180 days and 90 days before the LMP, not an etiologically significant window for congenital malformations. A null finding from the NCA indicates that the main findings were unaffected by residual confounding. 6. For outcomes that presented an increased risk, we will conduct a rule-out approach to explore the impact of unmeasured confounders (e.g., maternal smoking status). 7. As the study cohort included live births only, we will conduct a quantitative bias analysis based on the probabilistic method to address the impact of selection bias. |
| **Contacts and Locations:** | **Principal Investigator:** Ju-Young Shin, PhD, Sungkyunkwan University  **Locations:** Korea, Republic of, Sungkyunkwan University Suwon, Gyeonggi-do, Korea, Republic of, 16419  **Sponsors and Collaborators:** National Research Foundation of Korea |
| **Trial registration:** | **ClinicalTrials.gov Identifier:** NCT04856436 (Posted on April 23, 2021) |
