# Supplementary material for: First-trimester exposure to benzodiazepines and risk of congenital malformations in offspring: A population-based cohort study in South Korea
Source: PLoS Med. 2022 Mar 2;19(3):e1003945. doi: 10.1371/journal.pmed.1003945 (PMC8926183; doi:10.1371/journal.pmed.1003945)
Supplement: Attachment — Submitted filename: 1_Response letter.docx [file pmed.1003945.s013.docx]

**Editor and Reviewers’ Comments to Author:**

We thank the editors and reviewers for their helpful comments and suggestions. We believe that we have improved the manuscript substantially in response to their comments. In the following pages, we provide a point-by-point response to all comments raised.

**Requests from the editors:**

**Please remove the information on funding, competing interests and data availability from the title page. This information will appear in the article metadata in the event of publication, via entries in the submission form.**

Response: We moved the information to the Acknowledgement section on Page 25.

**In the abstract and throughout the text, please include p values alongside 95% CI, where available.**

Response: We added p-values alongside 95% CIs in the Abstract, Text, and Figures 1-5.

**At line 72, should that be "95% confidence intervals"?**

Response: We apologize for the mistake. We corrected it.

**Please quote aggregate demographic details for study participants in the abstract.**

Response: Thank you for your comment. To present demographics, we added information on the maternal age of the study cohort in the Abstract as below (Page 3, Line 50-52):

“Among a total of 3,094,227 pregnancies, 40,846 (1.3%) were exposed to benzodiazepines during the first trimester (mean [SD] age, 32.4 [4.1] years).”

**Please add a new final sentence to the "Methods and findings" subsection of your abstract, which should begin "Study limitations include ..." or similar, and should quote 2-3 of the study's main limitations.**

Response: We added limitations in the last sentence of the Abstract “Method and Findings” section as below (Page 3, Line 61-62):

“Study limitations include possible exposure misclassification, residual confounding, and restriction to live births.”

**At line 86, please begin the sentence "In this study, we found that ..." or similar.**

Response: We revised the sentence as below (Page 4, Line 64):

“In this large nationwide cohort study, we found that first-trimester benzodiazepine exposure is…”

**You mention a study protocol in the Methods section. If available, please include the document as an attachment, labeled "S1_Protocol" or similar, referred to in the text.**

Response: We attached “S1_Protocol” as a supplementary document as below:

**“S1_Protocol”**

**SUMMARY PROTOCOL**

| **Protocol Title:** | Maternal and Neonatal Outcomes Following Exposure to Benzodiazepines During Pregnancy |
| --- | --- |
| **Study Objective:** | To assess the association between benzodiazepine use in early pregnancy and the risk of congenital malformations |
| **Study type:** | Observational |
| **Study Design:** | A retrospective nationwide cohort study using the Health Insurance Review and Assessment (HIRA) database of South Korea, which covers the entire South Korean population. |
| **Study Population:** | Pregnant women who gave birth in South Korea between 2011 and 2018  **Sex/Gender:** Female  **Ages:** 20 years to 45 years (adult)  **Inclusion Criteria:**   - Pregnancies with live birth, 2011-2018 - Pregnancies linked to liveborn infants - Pregnancies aged 20-45 years at delivery   **Exclusion Criteria:**   - Pregnancies with a chromosomal abnormality - Pregnancies with exposure to known teratogenic drugs (e.g. antineoplastic agent, warfarin, lithium, isotretinoin, misoprostol, thalidomide) during the first trimester - Pregnancies with no benzodiazepine prescription during the first trimester, but with at least one benzodiazepine prescription during the 3 months before the pregnancy onset |
| **Intervention:** | Exposure to benzodiazepine during early pregnancy  **Groups:**  1) Pregnancies with exposure to benzodiazepines:  Women receiving at least one benzodiazepine prescription during the first trimester (first 90 days of pregnancy).  2) Pregnancies without exposure to benzodiazepines:  Women who did not receive a benzodiazepine prescription during the three months before the pregnancy onset through the end of the first trimester |
| **Sample Size:** | Based on the estimated number of live births during 2011-2018 from the Korean Statistical Information Service (KOSIS), we anticipated that our study cohort would include approximately 3 million pregnancies in this study. |
| **Study Endpoints:** | Risk of congenital malformations [Time Frame: from the birth date until up to 8 years, death, or study end date (Dec 31, 2019)]  Overall congenital malformations and organ-specific congenital malformations in infants, which are confirmed by diagnostic records in the HIRA database. All infants were followed up for at least one year. |
| **Statistical Methods:** | **Main analysis:**   - The balance of characteristics between the two groups will be evaluated using an absolute standardized difference (aSD), where a value >0.1 indicates a significant imbalance. - The absolute risks (per 1,000 pregnancies), risk differences, and unadjusted relative risks (RR) with 95% confidence interval (CI) for risk of major malformations were calculated, stratified by exposure to benzodiazepines. - Propensity score (PS) fine stratification method (PS weighting after fine stratification) will be used to control for potential confounders and the surrogates of potential confounders. - The adjusted RR with 95% CI will be estimated using a weighted generalized linear model (log-binomial).   **Subgroup analysis:**   - We will conduct seven pre-specified subgroup analyses as below: - Dose-response relationship - Duration of action (short-acting vs. long-acting) - Individual ingredients - Four maternal characteristics (potential risk factors for congenital malformations): maternal age, multiple pregnancy, epilepsy, and concomitant use of antidepressants   **Sensitivity analysis:**   1) Exposure will redefine as having two or more BZD prescriptions in the first trimester to minimize exposure misclassification. 2) The outcome will redefine as the presence of ≥2 diagnoses of congenital malformations to consider possible outcome misclassification. 3) We will restrict the study cohort to those who had underlying comorbidities related to the primary indication for benzodiazepines (e.g., bipolar disorder, depression/mood disorder, anxiety, sleep disorder, and gastrointestinal disease) to mitigate confounding by indication. 4) We will restrict the study cohort to nulliparous women to account for intra-individual correlations. 5) We will conduct a negative control analysis (NCA) by ascertaining exposure between 180 days and 90 days before the LMP, not an etiologically significant window for congenital malformations. A null finding from the NCA indicates that the main findings were unaffected by residual confounding. 6) For outcomes that presented an increased risk, we will conduct a rule-out approach to explore the impact of unmeasured confounders (e.g., maternal smoking status). 7) As the study cohort included live births only, we will conduct a quantitative bias analysis based on the probabilistic method to address the impact of selection bias. |
| **Contacts and Locations:** | **Principal Investigator:** Ju-Young Shin, PhD, Sungkyunkwan University  **Locations:** Korea, Republic of, Sungkyunkwan University Suwon, Gyeonggi-do, Korea, Republic of, 16419  **Sponsors and Collaborators:** National Research Foundation of Korea |
| **Trial registration:** | **ClinicalTrials.gov Identifier:** NCT04856436 (Posted on April 23, 2021) |

**Please highlight analyses that were not prespecified.**

Response: We attached the study protocol as a supplementary document “S1_Protocol.” All statistical analyses, including subgroup and sensitivity analyses, were performed as pre-defined or pre-specified. To clarify this, we added the following sentence in the Methods section as below (Page 11, Line 228-229):

“All analyses were conducted according to the pre-specified analysis plan (S1 Protocol).”

**Throughout the main text, please reformat the reference call-outs as follows: "... during pregnancy [9,10]. However, evidence ..." (noting the absence of spaces within the square brackets).**

Response: We re-formatted the reference callouts accordingly.

**In the reference list, please remove the copyright information from reference 9.**

Response: We removed the copyright information from reference #9.

**In reference 15, please make the journal name abbreviation "BMJ"; and for reference 20 use the journal name abbreviation "PLoS ONE"; and "JAMA" for reference 37.**

Response: We corrected those citations accordingly.

**Thank you for including a completed STROBE checklist. Please rename the attachment "S1_STROBE_Checklist" or similar, and refer to it by this label in the Methods section (main text).**

Response: We renamed the checklist as “S1_STROBE_Checklist”.

**Comments from academic editor:**

**This paper addresses a clinically relevant question and the methods seem generally sound. The lack of information on terminations of pregnancy is the major limitation.**

**I agree with the reviewer re the comment on inclusion of livebirths only. If there was any opportunity to do an additional analysis with stillbirth/miscarriage/terminations that would strengthen the study (if not possible this should be discussed as a limitation).**

Response: Thank you for your comment. We agree that the lack of information on pregnancy terminations is a limitation of our study, and acknowledged the limitation on page 24 of the manuscript. Our database does not contain information on gestational age for non-live births (i.e., stillbirth, miscarriage, terminations); it is not possible to analyze the risk of pregnancy terminations following medication use during pregnancy. To address this concern (i.e., to measure the impact of potential selection bias on our effect estimates [relative risks, RRs] due to the lack of information on pregnancy terminations), we conducted a quantitative bias analysis based on the probabilistic method (S2 Appendix describes in detail). This analytic approach has been generally used in previous studies when evaluating the risk of congenital malformation following maternal medication use in the absence of information on pregnancy terminations using the claims database [1-7].

As a result, the effect estimates (RRs) of congenital malformation in benzodiazepine users compared to non-users did not change dramatically, even under the assumption that the selection bias due to pregnancy termination was severe. Nonetheless, we discussed the limitation on page 24 and the Abstract “Methods and findings” section to clarify this limitation.

1 Huybrechts KF, et al. Antidepressant use in pregnancy and the risk of cardiac defects. N Engl J Med. 2014 Jun 19;370(25):2397-407. doi: 10.1056/NEJMoa1312828.

2 Bateman, et al. Statins and congenital malformations: cohort study. BMJ. 2015 Mar 17;350:h1035. doi: 10.1136/bmj.h1035.

3 Patorno E, et al. Lithium Use in Pregnancy and the Risk of Cardiac Malformations. N Engl J Med. 2017 Jun 8;376(23):2245-2254. doi: 10.1056/NEJMoa1612222.

4 Huybrechts KF, et al. Association of Maternal First-Trimester Ondansetron Use With Cardiac Malformations and Oral Clefts in Offspring. JAMA. 2018 Dec 18;320(23):2429-2437. doi: 10.1001/jama.2018.18307.

5 Zhu Y, et al. Oral fluconazole use in the first trimester and risk of congenital malformations: population based cohort study. BMJ. 2020 May 20;369:m1494. doi: 10.1136/bmj.m1494.

6 Patorno E, et al. Gabapentin in pregnancy and the risk of adverse neonatal and maternal outcomes: A population-based cohort study nested in the US Medicaid Analytic eXtract dataset. PLoS Med. 2020 Sep 1;17(9):e1003322. doi: 10.1371/journal.pmed.1003322. eCollection 2020 Sep.

7 Bateman, et al. Association of first trimester prescription opioid use with congenital malformations in the offspring: population based cohort study. BMJ. 2021 Feb 10;372:n102. doi: 10.1136/bmj.n102.

**An additional small point- could the authors explain rationale for excluding pregnancy in women <20y?**

Response: Adolescent pregnancies are more frequent in marginalized communities, commonly driven by poverty, low education, and low employment opportunities. Adolescent pregnancies are also more likely to have difficulty receiving proper antenatal care and engage in undesirable pregnancy behaviors, usually unmeasured in the healthcare database. Therefore, we excluded teenage pregnancies from our study cohort to minimize the potential of residual confounding.

**Comments from the reviewers:**

***** Reviewer #1:**

**Statistical review**

**This paper reports a retrospective cohort study assessing the association between prescription of benzodiazepine during first trimester and congenital malformations at birth. The results show a small but significant association. Many sensitivity results are provided that suggest the main results are robust.**

**I have some comments that I have listed below.**

**1. Abstract: I think it's fair to put that 'is associated' rather than 'may be associated' as long as association is not taken to be the same as causality. Would be good to emphasize the low absolute risk?**

Response: Thank you for your suggestion. We changed ‘may be associated’ to ‘is associated’ (Page 4, Line 64-65). Also, we emphasized the low absolute risk by adding the following sentence in the “Conclusion” sections of the Abstract (Page 4, Line 66) and main text (Page 25, Line 379-380):

“The absolute risks and population attributable fractions were modest.”

**2. Methods page 7 - please clarify somewhere (and in the title) that this is a retrospective cohort study.**

Response: We clarified that this study is a ‘retrospective’ cohort study on Page 7 (Line 125).

**3. Page 7 - why was the analysis restricted to live births? Is there no data on miscarriage/stillbirth? I see this is investigated in the supplementary material, although only in the direction of the main analysis result being underestimated. If the data is available then it might be informative to include a sensitivity analysis that includes miscarriage/stillbirth in the overall outcome of any abnormality.**

Response: Thank you for your comment. Our database does not contain information on gestational age for non-live births (i.e., miscarriage, stillbirth, termination). Thus, it is not possible to accurately measure medication use status during pregnancy and the outcome risk associated with non-live births. To address this concern (i.e., to measure the impact of potential selection bias on our effect estimates [relative risks] due to the lack of information on pregnancy terminations), we conducted a quantitative bias analysis (S2 Appendix describes in detail). This method has been generally used when evaluating the risk of congenital malformation in the absence of information on pregnancy terminations using the claims database [1-7].

As a result, the effect estimates (RRs) of congenital malformation in benzodiazepine users compared to non-users did not change dramatically, even under the assumption that the selection bias due to pregnancy termination was severe. Nonetheless, we discussed the limitation on page 24 and the Abstract “Methods and findings” section of the revised manuscript to clarify this limitation.

1 Huybrechts KF, et al. Antidepressant use in pregnancy and the risk of cardiac defects. N Engl J Med. 2014 Jun 19;370(25):2397-407. doi: 10.1056/NEJMoa1312828.

2 Bateman, et al. Statins and congenital malformations: cohort study. BMJ. 2015 Mar 17;350:h1035. doi: 10.1136/bmj.h1035.

3 Patorno E, et al. Lithium Use in Pregnancy and the Risk of Cardiac Malformations. N Engl J Med. 2017 Jun 8;376(23):2245-2254. doi: 10.1056/NEJMoa1612222.

4 Huybrechts KF, et al. Association of Maternal First-Trimester Ondansetron Use With Cardiac Malformations and Oral Clefts in Offspring. JAMA. 2018 Dec 18;320(23):2429-2437. doi: 10.1001/jama.2018.18307.

5 Zhu Y, et al. Oral fluconazole use in the first trimester and risk of congenital malformations: population based cohort study. BMJ. 2020 May 20;369:m1494. doi: 10.1136/bmj.m1494.

6 Patorno E, et al. Gabapentin in pregnancy and the risk of adverse neonatal and maternal outcomes: A population-based cohort study nested in the US Medicaid Analytic eXtract dataset. PLoS Med. 2020 Sep 1;17(9):e1003322. doi: 10.1371/journal.pmed.1003322. eCollection 2020 Sep.

7 Bateman, et al. Association of first trimester prescription opioid use with congenital malformations in the offspring: population based cohort study. BMJ. 2021 Feb 10;372:n102. doi: 10.1136/bmj.n102.

**4. Statistical methods - was a pre-specified analysis plan written? Propensity score matching has so many different methods to implement that it would be useful to know that the reported results were using the pre-planned method.**

Response: We apologize for causing confusion regarding a pre-specified analysis plan. To clarify the pre-specified analysis, we attached a supplementary document, “S1_Protocol,” developed before starting this study and registered in ClinicalTrials.gov (Identifier: NCT04856436, Posted on April 23, 2021). Moreover, we added the following sentence in the Methods section (Page 11, Line 228-229):

“All analyses were conducted according to the pre-specified analysis plan (S1 Protocol).”

Regarding the propensity score (PS) method used in this study, we implemented a “PS-based fine stratification approach,” which is one of the PS-based weighting methods [1,2]. This approach has been developed to minimize bias of measured confounding when exposure is infrequent; thus, is widely used in observational studies on pregnancy cohorts [3-10]. For this reason, we had chosen this PS approach as a pre-planned statistical method, and we clarified this in the document "S1_Protocol."

1 Desai RJ, et al. Epidemiology. A Propensity-score-based Fine Stratification Approach for Confounding Adjustment When Exposure Is Infrequent. 2017 Mar;28(2):249-257. doi: 10.1097/EDE.0000000000000595.

2 Desai RJ, et al. Alternative approaches for confounding adjustment in observational studies using weighting based on the propensity score: a primer for practitioners. BMJ. 2019 Oct 23;367:l5657. doi: 10.1136/bmj.l5657.

3 Huybrechts KF, et al. Antidepressant use in pregnancy and the risk of cardiac defects. N Engl J Med. 2014 Jun 19;370(25):2397-407. doi: 10.1056/NEJMoa1312828.

4 Bateman, et al. Statins and congenital malformations: cohort study. BMJ. 2015 Mar 17;350:h1035. doi: 10.1136/bmj.h1035.

5 Patorno E, et al. Lithium Use in Pregnancy and the Risk of Cardiac Malformations. N Engl J Med. 2017 Jun 8;376(23):2245-2254. doi: 10.1056/NEJMoa1612222.

6 Huybrechts KF, et al. Association of Maternal First-Trimester Ondansetron Use With Cardiac Malformations and Oral Clefts in Offspring. JAMA. 2018 Dec 18;320(23):2429-2437. doi: 10.1001/jama.2018.18307.

7 Zhu Y, et al. Oral fluconazole use in the first trimester and risk of congenital malformations: population based cohort study. BMJ. 2020 May 20;369:m1494. doi: 10.1136/bmj.m1494.

8 Patorno E, et al. Gabapentin in pregnancy and the risk of adverse neonatal and maternal outcomes: A population-based cohort study nested in the US Medicaid Analytic eXtract dataset. PLoS Med. 2020 Sep 1;17(9):e1003322. doi: 10.1371/journal.pmed.1003322. eCollection 2020 Sep.

9 Bateman, et al. Association of first trimester prescription opioid use with congenital malformations in the offspring: population based cohort study. BMJ. 2021 Feb 10;372:n102. doi: 10.1136/bmj.n102.

10 Huybrechts KF, et al. Maternal and fetal outcomes following exposure to duloxetine in pregnancy: cohort study. BMJ. 2020;368:m237. Published 2020 Feb 19. doi:10.1136/bmj.m237

**5. Page 9/10: How complete were covariates and, if there were non-negligible proportions of missing data, how were PS estimated in the presence of missing covariates?**

Response: Healthcare utilization databases contain complete information on all diagnoses, procedures, and medications recorded; thus, our covariates have no missing data. South Korea has a national health insurance system with a universal single-payer healthcare provider, covering 99% of the entire population [1]. Under the universal system, the HIRA data (our data) was generated in the process of reimbursing claims for healthcare services based on the fee-for-service model. Therefore, our data includes all information on demographics and healthcare utilization in the entire medical setting (e.g., inpatient, outpatient, emergency visit). We denoted this in “S1_STROBE_Checklist.”

1 Kim JA, et al. Towards Actualizing the Value Potential of Korea Health Insurance Review and Assessment (HIRA) Data as a Resource for Health Research: Strengths, Limitations, Applications, and Strategies for Optimal Use of HIRA Data. J Korean Med Sci. 2017 May;32(5):718-728. doi: 10.3346/jkms.2017.32.5.718.

**6. Page 10 - was the log-binomial model adjusted for the covariates or just matched on propensity score?**

Response: We implemented a “propensity score (PS)-based fine stratification approach” in our study [1]. This approach is one of the methods based on the PS, but simultaneously considers both ‘stratification’ and ‘weighting’ [1,2]. To implement this approach, we first estimated the PS for the use of benzodiazepines using a logistic regression model with a large number of covariates. Second, we excluded the individual pregnancies from non-overlapping regions of PS distribution. Third, we divided the exposed group into 50 strata based on calculated PS. Fourth, we calculated weights for the unexposed group using 50 strata of PS from the exposed group to balance the cohort characteristics among exposed and unexposed groups. Finally, we calculated adjusted relative risks using a weighted generalized linear model to account for weighting. Please see the two reference below for more information on this method.

1 Desai RJ, et al. Epidemiology. A Propensity-score-based Fine Stratification Approach for Confounding Adjustment When Exposure Is Infrequent. 2017 Mar;28(2):249-257. doi: 10.1097/EDE.0000000000000595.

2 Desai RJ, et al. Alternative approaches for confounding adjustment in observational studies using weighting based on the propensity score: a primer for practitioners. BMJ. 2019 Oct 23;367:l5657. doi: 10.1136/bmj.l5657.

**7. Page 10/11 - I do not follow the negative control analysis, is this comparing women exposed 180-90 days before vs those never exposed at any point? I would rephrase the statement on line 238 to be less definitive, e.g. 'If the main analysis were subject to residual confounding then we would expect the negative control analysis to give similar results'?**

Response: We thank the reviewer for the opportunity to clarify this. In the negative control analysis, we compared two following groups: a) pregnancies exposed to benzodiazepines between 180 days and 90 days before the last menstrual period, which is not an etiologically relevant window for congenital malformation (negative exposure control); b) pregnancies not exposed to benzodiazepines in the first trimester. For a more detailed explanation, we modified the sentence as below (Page 11, Line 219-224):

“Fifth, we conducted a negative control analysis by comparing negative exposure control (defined as pregnancies exposed to benzodiazepines between 180 days and 90 days before the last menstrual period, which is not an etiologically relevant window for congenital malformations) with the reference group in the main analysis (pregnancies not exposed to benzodiazepines in the first trimester).”

Also, as you suggested, we rephrased the statement to more definitive as below (Line 224-5):

“If the main finding is subject to residual confounding, we can expect a non-null result from the negative control analysis.”

**8. Results - just as a suggestion, it would be of interest to estimate the proportion of abnormalities that are explained by benzodiazepine (i.e., the population attributable fraction).**

Response: Thank you for your comment. As you suggested, we added the population attributable fractions (PAFs) for all types of congenital malformation (Figure 1 & 2). We also added the statement regarding PAFs in the Methods and the Results sections:

“Methods (Page 10, Line 192-197)”

“Moreover, we calculated the population-attributable fractions (PAFs) to estimate the impact on an outcome of exposure in the study population [30]. In this study, PAF means the fractions of overall or individual congenital malformations in pregnant women attributable to benzodiazepines. We calculated PAF as (O-E)/O, where O is the observed number of outcomes and E is the expected number of outcomes under no exposure [31].”

“Results (Page 14, Line 249-250)”

“The PAFs of overall malformations and heart defects were 0.36% and 0.55%, respectively.”

**Fig 1.** Absolute and relative risks of congenital malformations in infants following maternal exposure to benzodiazepines during the first trimester. Abbreviations: CI, confidence interval; PS, propensity score; PAF, population attributable fraction; *RD_1000_, risk difference per 1,000 births.

**Fig 2.** Risks of individual categories of heart and digestive system defects in infants following maternal exposure to benzodiazepines during the first trimester. Abbreviations: CI, confidence interval; PAF, population attributable fraction; PS, propensity score; *RD1000, risk difference per 1,000 births.

***** Reviewer #2:**

**The manuscript does an interesting job generating scientific evidence on a significant topic on human health. The aim of the manuscript was to evaluate the association between first trimester benzodiazepine use and the risk of major congenital malformations. For that, authors have acceded to Korea's nationwide healthcare database, and conducted a population-based cohort study of women who gave birth during 2011-2018 and their live-born infants.**

**The manuscript it is well written, its design, its methodology and statistical approach applied on data it is sound according to statistical analysis are applying actually in this field.**

Response: We thank Reviewer #2 for the positive feedback regarding our study.

**General comments:**

**The main concern it is about how to interpret "congenital malformations" as main outcome. Congenital malformations are a wide range of heterogeneous conditions with highly heterogeneous causes.**

**It would be very useful if the manuscript could be presented disaggregating the risks over a reduced group of specific categories of congenital malformations. Probably if authors could focus on a list of specific categories (or groups of categories), such as there are made for heart defects, the manuscript will produce a more precise information. Authors show this kind of results but in supplementary tables. Please consider if it could be possible to select a group of specific categories (or group of) and them present it as main results.**

Response: We thank the reviewer for the thoughtful suggestion. As you suggested, we presented the result that describes the risks of specific categories of congenital malformations as the main result (**Figure 2**) of the revised manuscript. We explored the risks of individual categories of heart defects and the digestive system, as previous studies reported that these specific outcomes might be associated with benzodiazepines. Due to the small number of events, other organ-specific outcomes were not analyzed as individual sub-categories.

**-When authors have defined the exposition, and therefore exposed and unexposed group, have been women in the exposed group exclusively exposed to benzodiazepines? or they could be exposed to another drug also? the same question is valid for the unexposed group, were those women exposed to other drug or none drug?**

**Authors would agree that theoretically there are four possibilities of exposition:
a-exposed only to Benzodiazepines
b-exposed to benzodiazepines + other drug
c-exposed to other/s drug/s
d-none drug exposed during pregnancy**

**Please add information about how frequent it is the exposition to other drugs in this groups. Since depending on how the relative risk is established with any combination of these groups, this RR could be interpreted in different ways. Please feel free to consider the following reference if it is useful: DOI: 10.1371/journal.pone.0046626**

Response: Thank you for your comment. As numerous other cohort studies have done, we defined benzodiazepine exposure with or without other medication use [1-8]. We thoroughly reviewed the article (DOI: 10.1371/journal.pone.0046626) you mentioned above and pondered the potential impact of any other drugs on our effect estimates (relative risks). After all consideration, we reached the conclusion that the approach suggested by the article is not suitable to apply in our cohort study.

In general, case-control studies are vulnerable to selection bias and information bias (recall bias). Those biases are a particular problem inherent in a case-control design, and they give non-comparability between ‘cases’ and ‘controls.’ Selection bias in a case-control design occurs when subjects for the 'control' group are not truly representative of the population that produced the cases (for example, the drug-exposed individuals are more likely to be selected in the 'case' group).

The article (DOI: 10.1371/journal.pone.0046626) addresses the potential selection and recall biases when conducting a “case-control study” using a “surveillance program” for birth defects. The study evaluated the potential impact of those biases by applying different definitions and selections of ‘control’ groups in the case-control study. To be concluded, the study suggested that the “Only-Exposed Cases” design (restricting case and control to pregnant women who were exposed to any type of medication during pregnancy) is one of the approaches that minimize the selection and recall bias. This approach corresponds the method suggested by the reviewer.

However, we conducted a cohort study design, which includes the entire population of South Korea; thus, there was no selection bias in our study. In addition, there was no information (recall) bias, as we used a healthcare utilization database, not a surveillance program based on the reporting system. Furthermore, we made several efforts to minimize confounding bias caused by other drug use. First, we excluded pregnant women prescribed known teratogenic medication from the study cohort. Second, we adjusted a large number of covariates, including 19 concomitant medications and the number of distinct prescription drugs (excluding benzodiazepines).

As the reviewer suggested, we calculated the frequency of the four expositions. The proportion of exposure to other drugs differed among the exposed and unexposed groups as below:

|  | **Benzodiazepine-exposed**  **(n=40,846)** | | **Unexposed**  **(n=3,053,381)** | |
| --- | --- | --- | --- | --- |
|  | **a-exposed to benzodiazepines only** | **b-exposed to benzodiazepines and other drugs** | **c-exposed to other drugs only** | **d-not exposed to any drugs** |
| **n (%)** | 9,891 (24.2) | 30,955 (75.8) | 1,003,638 (32.9) | 2,049,743 (67.1) |

However, after applying the propensity score-based stratification and weighting approach, the distributions of other drugs other than benzodiazepines were well-balanced (aSD<0.1) as below (Table 1):

|  | **Unadjusted** | | | | |  | **PS adjusted^*^** | | | | |
| --- | --- | --- | --- | --- | --- | --- | --- | --- | --- | --- | --- |
|  | **Benzodiazepines (n=40,846)** | | **Unexposed (n=3,053,381)** | | **aSD^†^** |  | **Benzodiazepines (n=40,844)** | | **Unexposed (n=3,053,207)** | | **aSD^†^** |
| **Concomitant medications, n (%)** |  |  |  |  |  |  |  |  |  |  |  |
| Antidepressants | 6,910 | (16.9) | 43,245 | (1.4) | 0.56 |  | 6,909 | (16.9) | 470,554 | (15.4) | 0.04 |
| Anxiolytics | 2,744 | (6.7) | 98,944 | (3.2) | 0.16 |  | 2,743 | (6.7) | 207,339 | (6.8) | 0.00 |
| Hypnotics | 3,129 | (7.7) | 19,387 | (0.6) | 0.36 |  | 3,128 | (7.7) | 198,914 | (6.5) | 0.04 |
| Barbiturates | 967 | (2.4) | 41,455 | (1.4) | 0.07 |  | 967 | (2.4) | 79,843 | (2.6) | 0.02 |
| Anticonvulsants | 1,508 | (3.7) | 21,158 | (0.7) | 0.21 |  | 1,508 | (3.7) | 107,435 | (3.5) | 0.01 |
| Antipsychotics | 2,126 | (5.2) | 5,842 | (0.2) | 0.31 |  | 2,125 | (5.2) | 113,254 | (3.7) | 0.07 |
| Stimulants | 112 | (0.3) | 411 | (0.0) | 0.07 |  | 112 | (0.3) | 6,767 | (0.2) | 0.01 |
| Opioid analgesics | 17,486 | (42.8) | 602,813 | (19.7) | 0.51 |  | 17,485 | (42.8) | 1,428,633 | (46.8) | 0.08 |
| Non-insulin antidiabetic agents | 334 | (0.8) | 10,448 | (0.3) | 0.06 |  | 334 | (0.8) | 26,611 | (0.9) | 0.01 |
| Insulins | 262 | (0.6) | 9,811 | (0.3) | 0.05 |  | 262 | (0.6) | 20,127 | (0.7) | 0.00 |
| Antihypertensives | 4,097 | (10.0) | 44,679 | (1.5) | 0.37 |  | 4,096 | (10.0) | 287,528 | (9.4) | 0.02 |
| Non-steroidal anti-inflammatory drugs | 26,474 | (64.8) | 1,269,496 | (41.6) | 0.48 |  | 26,473 | (64.8) | 2,106,157 | (69.0) | 0.09 |
| Triptans | 445 | (1.1) | 7,030 | (0.2) | 0.11 |  | 445 | (1.1) | 35,619 | (1.2) | 0.01 |
| Lipid lowering drug | 414 | (1.0) | 8,629 | (0.3) | 0.09 |  | 414 | (1.0) | 31,005 | (1.0) | 0.00 |
| Anti-thyroid drugs | 360 | (0.9) | 17,774 | (0.6) | 0.04 |  | 360 | (0.9) | 28,429 | (0.9) | 0.01 |
| Thyroid hormones | 1,594 | (3.9) | 112,391 | (3.7) | 0.01 |  | 1,594 | (3.9) | 121,123 | (4.0) | 0.00 |
| Systemic corticosteroids | 10,386 | (25.4) | 466,690 | (15.3) | 0.25 |  | 10,386 | (25.4) | 828,069 | (27.1) | 0.04 |
| Azoles | 12,161 | (29.8) | 732,996 | (24.0) | 0.13 |  | 12,160 | (29.8) | 928,981 | (30.4) | 0.01 |
| Fertility drugs | 2,774 | (6.8) | 98,401 | (3.2) | 0.16 |  | 2,774 | (6.8) | 237,642 | (7.8) | 0.04 |
| **Number of distinct prescription drugs,**  **excluding benzodiazepines, mean (SD)** | 16.4 | (12.0) | 10.1 | (8.9) | 0.60 |  | 16.4 | (12.0) | 17.0 | (11.8) | 0.05 |

**Abbreviations**: aSD=absolute standardized difference. ^*^To account for PS, unexposed observations were weighted using the distribution of the exposed observations among the 50 PS strata. Observations from non-overlapping regions of the PS distributions were trimmed. ^†^The value >0.10 indicates an imbalance between the two groups.

In addition, to evaluate whether unmeasured confounding bias remains, we conducted various sensitivity analyses, including a negative control and a rule-out approach analysis. All results (Figure 5 and S4-S6; S1 Appendix) showed that our findings were not affected mainly by confounding bias.

1 Huybrechts KF, et al. Antidepressant use in pregnancy and the risk of cardiac defects. N Engl J Med. 2014 Jun 19;370(25):2397-407. doi: 10.1056/NEJMoa1312828.

2 Bateman, et al. Statins and congenital malformations: cohort study. BMJ. 2015 Mar 17;350:h1035. doi: 10.1136/bmj.h1035.

3 Patorno E, et al. Lithium Use in Pregnancy and the Risk of Cardiac Malformations. N Engl J Med. 2017 Jun 8;376(23):2245-2254. doi: 10.1056/NEJMoa1612222.

4 Huybrechts KF, et al. Association of Maternal First-Trimester Ondansetron Use With Cardiac Malformations and Oral Clefts in Offspring. JAMA. 2018 Dec 18;320(23):2429-2437. doi: 10.1001/jama.2018.18307.

5 Zhu Y, et al. Oral fluconazole use in the first trimester and risk of congenital malformations: population based cohort study. BMJ. 2020 May 20;369:m1494. doi: 10.1136/bmj.m1494.

6 Patorno E, et al. Gabapentin in pregnancy and the risk of adverse neonatal and maternal outcomes: A population-based cohort study nested in the US Medicaid Analytic eXtract dataset. PLoS Med. 2020 Sep 1;17(9):e1003322. doi: 10.1371/journal.pmed.1003322. eCollection 2020 Sep.

7 Bateman, et al. Association of first trimester prescription opioid use with congenital malformations in the offspring: population based cohort study. BMJ. 2021 Feb 10;372:n102. doi: 10.1136/bmj.n102.

8 Huybrechts KF, et al. Maternal and fetal outcomes following exposure to duloxetine in pregnancy: cohort study. BMJ. 2020;368:m237. Published 2020 Feb 19. doi:10.1136/bmj.m237

**Minor comments or questions:**

**-Have authors considered to obtain Population attributable fraction as an indicator of benzodiazepines use's impact?**

Response: We thank Reviewer #2 for this suggestion. We added the population attributable fractions (PAFs) for all types of congenital malformation (Figure 1 & 2). Please refer to our response for a similar comment #8 by Reviewer #1.

**-When have mentioned covariates: have you information related to socioeconomic status, socioeconomic conditions, maternal education or similar? since these are closely related to the occurrence of specific birth defects, such as oral cleft, among others.**

Response: Thank you for your comment. Our database has no information on maternal education or socioeconomic status (SES). As a proxy of SES, we included medical aid beneficiaries (bottom 3% of income level in South Korea) as a covariate. Although more detailed information on SES is unavailable, we believe the covariate adjusts the SES-related effect.

**-When the** **relative risks for overall congenital malformations were obtained, were the congenital heart defects excluded?**

Response: Overall congenital malformations include all types of organ-specific malformation. Thus, heart defects were included when estimating the relative risks for overall malformations.

**-Have information on prenatal diagnosis for congenital malformation on these pregnancies? could this be another source of bias?**

Response: Thank you for your comment. If a pregnant woman is diagnosed with a congenital malformation in prenatal care, her pregnancy will end with live or non-live birth. In the case of live birth, we can capture the outcome in infant claims. In the case of non-live birth, we cannot obtain information on its gestational age; thus, we cannot evaluate the outcome risk associated with non-live birth. To address this issue, we conducted a quantitative bias analysis (S2 Appendix describes in detail). For more information, please refer to our response to comment #3 by Reviewer #1.

***** Reviewer #3:**

**This is an interesting manuscript with a purpose to "evaluate the association between first-trimester benzodiazepine use and the risk of major congenital malformations." This was a retrospective cohort study using a national database (the Health Insurance Review and Assessment Service (HIRA) database which covers approximately 99% of the South Korean population).**

**1. In the abstract "We determined the relative risks (RRs) and confidential internal (CI) of overall congenital malformations and..." Should this be confidence interval?**

Response: We apologize for this mistake. We made the correction.

**2. In the Introduction "The worldwide prevalence of benzodiazepines during pregnancy is approximately 2%,(4) and" Should this be benzodiazepine use?**

Response: Thank you for pointing this out. We changed “benzodiazepines” to “benzodiazepine use” (Page 6, Line 95).

**3. For Reference #22 do the authors have the journal and identifying information on where this reference was published? Do the authors have data on the validity of prescription drug entry into the database? Could the authors supply more information about how the data on prescription drugs are entered into the database and its reliability?**

Response: Reference #22 is a national report published by Health Insurance Review and Assessment Service (HIRA), and it is currently posted on the “HIRA OAK (Open Access Korea) Repository” website (Ref: Park E et al. Report of the evaluation for the validity of discharged diagnoses in Korean Health Insurance database. Health Insurance Review and Assessment Service (2017). <https://repository.hira.or.kr/handle/2019.oak/730>). Although it is not published in journal, it is issued by a credible national institution of South Korea.

Regarding the second and third questions, there is no data on the validity of prescription drug entry yet. However, all healthcare service providers in South Korea submit a claim on prescription drugs to HIRA to be reimbursed. They submit detailed information on drug prescriptions, including drug ingredients, the date of prescription/dispensing, days of supply, the total quantity of supply, dose, etc. Then, HIRA reviews whether the submitted claims are within the scope of approval and makes reimbursement decisions. The HIRA database is generated in this process of reimbursement, which is part of the government’s health insurance system; therefore, the data entered into the HIRA database are highly reliable and valid.

***** Reviewer #4:**

**This is a well-designed retrospective cohort study of Korean insurance data that assesses the impact of first-trimester exposure of benzodiazepines via active prescriptions on the risk of congenital malformations. The authors found a statistically significant difference in rate of overall and cardiac anomalies. The analysis for these outcomes is robust, including adjusting for 45 confounders. Though the findings are statistically significant, the increase is nominal and not likely clinically relevant, so I would recommend a tempering of language used with suggestions below.**

Response: We thank the reviewer #4 for this positive feedback regarding our study.

**Abstract:**

**1. Line 81: Would say "suggesting" rather than "indicating" as the incremental increase in adjusted RR is modest at best.**

Response: Thank you for this comment. We changed the wording as suggested (Page 3, Line 58).

**2. Line 81: I would make sure to clarify the degree of significance of the findings such as a "small but significant."**

Response: We agree with the reviewer’s comment, and we revised the sentence as follows (Page 3, Line 58):

“A small but significant increase in risk for overall and heart defects was detected with several specific agents…”

**3. Line 87-90: I would go so far as to say that in most cases "****the benefit of benzodiazepines is likely to outweigh the nominal increased risk" and that the lowest effective dose should be prescribed to "minimize risk"**

Response: Thank you for your suggestion. We agree that we should emphasize the benefits of benzodiazepines as the observed risk was small. Thus, we revised the sentence in the “Conclusion” section of the Abstract accordingly (Page 4, Line 66-69). Still, as our results alone are insufficient to conclude that the benefits of benzodiazepines ‘outweigh’ the risks, we slightly mitigated the expression while reflecting the comment as below:

“The absolute risks and population attributable fractions were modest. The benefits of benzodiazepines for their major indications must be considered despite the potential risks; if their use is necessary, the lowest effective dosage should be prescribed to minimize the risk.”

We also revised the “Conclusion” section of the main text as below (Page 24, Line 379-384):

“However, the absolute risks and population attributable fractions were modest. Our findings suggest that the benefits of benzodiazepines for their major indications must be considered despite the potential risks. Nonetheless, to minimize the potential risk, alternative non-pharmacological strategies could be considered for managing anxiety and insomnia during pregnancy; if benzodiazepines are necessary, the lowest effective dosage should be prescribed during early pregnancy.”

**Author summary:**

**1. Line 102-103: Suggest "slightly" as opposed to "particularly" and "suggesting a" instead of "indicating the"**

Response: We changed the wording as suggested (Line 81-82).

**2. Line 104-: Suggest "a small but significant increased risk"**

Response: We revised the sentence as suggested (Line 83).

**Methods:**

**1. Line 148 and 153: Why do the database dates and dates of the cohort differ?**

Response: Our cohort included all pregnancies resulting in live births from 2011 to 2018 (identified based on the delivery date). Within this population, exposure was assessed during pregnancy, outcomes were evaluated after delivery, and cohort baseline characteristics were measured before and during pregnancy. Thus, to determine exposure and baseline characteristics before and during pregnancy (i.e., before the delivery date), we needed to review data from 2009 to 2018. In addition, to follow-up infants at least one year, we need to review data up to 2019. Taken together, considering the exposure, outcome, and covariate assessment window, the data period (2009-2019) had to be longer than the cohort entry period (2011-2018).

**2. Line 156: What is the justification for this age range selection?**

Response: We restricted our study cohort to women aged 20-45, excluding teenage pregnancies (<20 years) and >45 years of age. Teenage pregnancies are more frequent in marginalized communities, commonly driven by poverty, low education, and low employment opportunities. Thus, they are more likely to have difficulty receiving proper antenatal care and engage in undesirable behaviors, usually unmeasured in the healthcare database. Women>45 years of age had little chance of natural pregnancies. Therefore, we excluded women of that age range to minimize possible residual confounding, which is unpredictable.

**3. Line 156-160: Was the possibility of substance use disorder also considered and controlled for? (e.g., not prescription-based drug use)**

Response: To control substance use disorders (ICD10: F10-F19), we included alcohol or drug dependence (ICD10: F10-F16, F18-F19, Z71.4, Z71.5, Z72.1, Z72.2) and tobacco dependence (ICD10: F17, Z71.6, Z72.0) as potential confounders. Below is the number of pregnancies with alcohol or drug dependence and tobacco dependence among benzodiazepine-exposed and unexposed pregnancies, also described in Table 1 as below:

|  | **Unadjusted** | | | | |  | **PS adjusted^*^** | | | | |
| --- | --- | --- | --- | --- | --- | --- | --- | --- | --- | --- | --- |
|  | **Benzodiazepines (n=40,846)** | | **Unexposed (n=3,053,381)** | | **aSD^†^** |  | **Benzodiazepines (n=40,844)** | | **Unexposed (n=3,053,207)** | | **aSD^†^** |
| Alcohol or drug dependence | 341 | (0.8) | 1,520 | (0.0) | 0.12 |  | 341 | (0.8) | 19,267 | (0.6) | 0.02 |
| Tobacco dependence | 2 | (0.0) | 36 | (0.0) | 0.01 |  | 2 | (0.0) | 174 | (0.0) | 0.00 |

**Abbreviations**: aSD=absolute standardized difference. ^*^To account for PS, unexposed observations were weighted using the distribution of the exposed observations among the 50 PS strata. Observations from non-overlapping regions of the PS distributions were trimmed. ^†^The value >0.10 indicates an imbalance between the two groups.

**4. Recommend a statistician review the analyses performed though seem appropriate.**

Response: Thank you for your suggestion. Our manuscript received a review from Reviewer #1, a statistician. Please refer to ‘Reviewer #1’ comments and our responses to them.

**Results/Discussion:**

**1. Line 276/308/337: Again suggest "small but significant(ly)"**

Response: We revised the sentence as suggested (Line 272/303/333).

**2. Line 311-312: I do not understand what this line means or what data this 63% comes from.**

Response: For all analyses, we presented a point estimate along with its 95% CI, which are informative measures as they provide information on the magnitude of effect and degree of precision of the estimate. Our interpretation is based on the upper limit of 95% CI from the main findings. As shown in Figure 1, among the organ-specific malformations, the upper limit of the 95% CI was highest in the abdominal wall defects (RR 1.08, 95% CI 0.72-1.63). It implies that the maximum observed risk was 63% under the confidence level of 95%; that is, we could rule out a more than 63% increase in the risk for all organ-specific malformations. To clarify this, we revised the sentence as below (Page 21, Line 305-308):

“Based on the upper limit of the 95% CI from adjusted estimates, the maximum observed risk was 63% for the abdominal wall defects (RR 1.08, 95% CI 0.72-1.63); thus, we could rule out the possibility of a more than 63% increase in the risk for all organ-specific malformations.”
