# Supplementary material for: First-trimester exposure to benzodiazepines and risk of congenital malformations in offspring: A population-based cohort study in South Korea
Source: PLoS Med. 2022 Mar 2;19(3):e1003945. doi: 10.1371/journal.pmed.1003945 (PMC8926183; doi:10.1371/journal.pmed.1003945)
Supplement: Attachment — Submitted filename: 1_Response letter_R2.docx [file pmed.1003945.s014.docx]

**Editor and Reviewers’ Comments to Author:**

We thank the editors and reviewers for their helpful comments and suggestions. We believe that we have improved the manuscript in response to their comments. In the following pages, we provide a point-by-point response to each comment.

**Requests from Editors:**

**Regarding the data statement, thank you for including a web address for inquiries about data (https://opendata.hira.or.kr). Readers outside Korea may find this site difficult to navigate, and we ask you to substitute a more specific web address, e.g.,** [**https://www.data.go.kr/en/index.do**](https://www.data.go.kr/en/index.do)**.**

Response: Thank you for your comment. We substituted it as suggested as below:

“However, the data are available from the HIRA on reasonable request for researchers who meet the criteria for access to confidential data (<https://www.data.go.kr/en/tcs/eds/selectCoreDataView.do?coreDataInsttCode=B551182&coreDataSn=1&searchCondition2=coreDataNmEn&searchKeyword2=>).”

**Please remove "Association between" from the title; and add "... in Korea" at the end, or make a similar amendment to indicate where the study was done.**

Response: We revised the title as requested:

“First-trimester Exposure to Benzodiazepines and Risk of Congenital Malformations in Offspring: A Population-Based Cohort Study in South Korea”

**We suggest indicating the end of the follow-up period in the abstract.**

Response: We added the below sentence in the Abstract section to indicate the end of the follow-up (Page 3, Line 49-50):

“Infants were followed from birth to death or the 31 December 2019, whichever came first (up to 8 years of age).”

**At line 41, we suggest amending the wording to "... evidence about possible teratogenicity is equivocal".**

Response: We revised the wording as suggested (Page 3, Line 41-2).

**At line 46, would "The exposure ..." be preferable to "Intervention"?**

Response: We changed the wording as suggested (Page 3, Line 46).

**At line 48, "confidence intervals", we imagine.**

Response: We apologize for the mistake. We corrected it.

**At line 64 (abstract) and any similar instances where you discuss observations, please make that "... was associated".**

Response: Thank you for pointing this out. We corrected it and reviewed it throughout the manuscript.

**At line 78, we suggest "... we found a small increased risk".**

Response: We revised it as suggested.

**Please remove the information about data availability, funding and competing interests from the end of the main text. In the event of publication, this information will appear in the article metadata, via entries in the submission form. A brief "Acknowledgements" section is permissible here, excluding this information.**

Response: We remove the information about data availability, funding, and competing interests from the main text.

**Throughout the manuscript, including the figures, please substitute "p<0.001" for "p<0.0001" and any other p values less than 0.001.**

Response: We substituted "p<0.001" for any p-values less than 0.001 throughout the manuscript and all figures.

**Comments from Reviewers:**

***** Reviewer #1:**

**Thank you to the authors for doing an excellent revision that addressed all my previous comments well. I particularly appreciated the additional sensitivity analysis in appendix S2.**

Response: We thank Reviewer #1 for the positive feedback.

***** Reviewer #2:**

**Questions were properly answered**

Response: We thank Reviewer #2 for the positive feedback.
